# Identification of Natural Flavonoids Targeting PLK-1 as Potential Anti-Metastatic Agents: A Computational Approach

**DOI:** 10.3390/ijms27156821

**Published:** 2026-07-29

**Authors:** Yudith Cañizares-Carmenate, Erix W. Hernández-Rodríguez, Yunier Perera-Sardiña, Dina B. Aguado-Herrera, Roberto Díaz-Amador, Francisco Torrens, Juan A. Castillo-Garit

**Affiliations:** 1 Laboratorio de Bioinformática y Química Computacional, Departamento de Medicina Traslacional, Facultad de Medicina, Universidad Católica del Maule, Talca 3460000, Chile; ehernandez@ucm.cl (E.W.H.-R.); rodiaz@ucm.cl (R.D.-A.); 2 Laboratory of Pharmacology and Physiology, Department of Basic Biomedical Sciences, Faculty of Health Sciences, University of Talca, Talca 3460000, Chile; yunier.perera@utalca.cl; 3 Unit of Computer-Aided Molecular “Biosilico” Discovery and Bioinformatic Research (CAMD-BIR Unit), Departamento de Farmacia, Facultad Química y Farmacia, Universidad Central ‘’Marta Abreu” de Las Villas, Santa Clara 54830, Villa Clara, Cuba; daguado@uclv.cu; 4 Institut Universitari de Ciència Molecular, Universitat de València, Edifici d’Instituts de Paterna, P.O. Box 22085, 46071 Valencia, Spain; francisco.torrens@uv.es; 5 Instituto Universitario de Investigación y Desarrollo Tecnológico (IDT), Universidad Tecnológica Metropolitana, Ignacio Valdivieso 2409, San Joaquín, Santiago 8940577, Chile

**Keywords:** cancer treatment, docking, molecular dynamics simulation, polo-like kinase-1 inhibitor, virtual screening

## Abstract

This study combines ligand- and structure-based *in silico* strategies to predict the inhibitory activity of natural flavonoids on the Polo-Like Kinase-1 (PLK-1) enzyme as candidate anticancer agents. This enzyme participates in mitosis and is overexpressed in cancer cells. Furthermore, it has been shown to have important implications for tumor metastasis, and its inhibitors are attractive starting points for drug development. First, classification models are developed using linear discriminant analysis and a multilayer perceptron neural network. Models with accuracy greater than 80%, validated using standard statistical performance metrics and applicability domain, are used for virtual screening identifying four compounds as potential antitumor drugs. Subsequently, the identified compounds are evaluated using a molecular docking methodology to verify their binding mode and interactions with the catalytic domain of PLK-1. Finally, the integration of molecular dynamics simulations, at 300 ns, with Molecular Mechanics/Generalized Born Surface Area (MM/GBSA) thermodynamic calculations demonstrates that the hydroxylation pattern of ring B in the flavonol scaffold is the fundamental chemical-structural determinant of electrostatic interactions and the architecture of water-mediated networks. Among the evaluated flavonoids, myricetin showed the most favorable overall computational profile, including the highest virtual-screening score and the most favorable mean MM/GBSA estimate, supporting its prioritization for experimental evaluation as a potential PLK-1 inhibitor. The integration of these approaches offers a robust methodological framework for proposing candidates with a higher probability of success, in subsequent stages of experimental validation, reducing time and costs in the early stages of drug development.

## 1. Introduction

Cancer is a multifaceted disease that represents one of the greatest challenges to global public health. Its incidence is steadily rising, affecting millions of people. This disease is characterized by the transformation of normal cells into tumor cells, which is due to progressive accumulations of mutations in the different phases of cell division. Tumor cells multiply rapidly and spread beyond their normal limits and can invade adjacent parts of the body or spread to other organs, a process called metastasis [1,2,3].

According to the World Health Organization (WHO), in the last 20 years this disease has become one of the leading causes of mortality, causing more than 10 million deaths annually worldwide [1]. Unfortunately, several items make this disease dangerously prevalent: risk factors such as constant exposure to environmental carcinogens, toxic habits such as smoking and alcohol consumption, unhealthy diets, physical inactivity, obesity, as well as genetic components that predispose certain individuals and immunosenescence associated with the aging population, among others [4]. These variables not only underscore the need for robust prevention strategies but also highlight the urgency of innovation in the therapeutic field.

In this context, the Polo-Like Kinase-1 (PLK-1) enzyme has become an attractive target for the search for drugs that slow down the progression of the disease. This enzyme belongs to the serine/threonine kinase family and is an important regulator of mitotic progression [5,6,7,8,9], which contributes to the process of cell entry into mitosis, specifically in the G2-M phase transition [7,10,11,12]; it is also the most investigated member of its family, since it is highly expressed in proliferating cells and overexpressed in many cancers, thus representing an attractive target for therapeutic development against this disease [13,14].

The polo-like mitotic regulatory kinase-1 (called polo) gene was identified 30 years ago in *Drosophila melanogaster* [15]. It is made up of two domains: (1) the polo-box domain (PBD) at the C-terminal end, made up of two phosphopeptide binding sites, whose function is the correct subcellular localization of PLK-1, since they bind to the proteins that make up the centrosome and the kinetochores [16] and (2) the catalytic domain (CD) at the N-terminus, where its kinase function takes place, as well as an inter-domain linker (IDL) [17,18,19].

The PLK-1 enzyme has crucial roles in multiple aspects of mitosis [20], including mitotic entry, centrosome maturation, bipolar spindle assembly [21,22], chromatin segregation [23], G2/M phase transition, rupture nuclear envelope, localization of kinetochores, DNA replication, fragmentation of the Golgi apparatus, activation of APC/C by phosphorylation of early mitotic inhibitor 1 (Emi1), and activation of the CDK1-Cyclin B complex in M phase, exit mitotic and cytokinesis [7,8,12,22,24,25,26,27]. In addition to regulatory roles related to the initiation of mitosis, PLK-1 was found to be specifically involved in autophagy [28], apoptosis, and cytokine signaling [18,29].

Due to the excellent results with the antitumor effect both *in vitro* and *in vivo* by inhibiting this enzyme, much attention has been paid to its characterization, and even more to the synthesis or identification of its inhibitors [13]. Research on PLK1 inhibitors has been an active area in the development of oncology treatments, primarily focusing on catalytic domain inhibitors such as volasertib and onvansertib. However, the number of inhibitors specifically approved for cancer remains limited. So far, there have been advancements in clinical trials, but many of these compounds are still under investigation or have not been approved for widespread use due to a variety of adverse effects [30,31].

Natural compounds, particularly flavonoids, represent a valuable source of bioactive molecules with anticancer potential due to their ability to modulate multiple cells signaling pathways [32,33,34,35,36,37,38]. Previous studies have demonstrated that various flavonoids can act as kinase inhibitors, reducing tumor proliferation and promoting programmed cell death [39,40].

In spite of the progress in this area, pharmaceutical research is highly complex, as it faces challenges ranging from long development times to high costs and a significant risk of failure [41]. To address these limitations, computational approaches have emerged as valuable tools for the early identification and prioritization of promising drug candidates. These methods leverage complementary sources of information, including ligand structure, target structure, and molecular interactions, to support rational drug discovery. Their integration enables the development of increasingly robust predictive models, facilitating the generation of experimentally testable mechanistic hypotheses. In turn, this reduces unsuccessful experimental efforts and accelerates the drug discovery cycle [42,43,44]. The present work proposes an *in silico* strategy, which combines structure-activity relationship methods with molecular docking and molecular dynamics to identify natural compounds with potential antitumor activity.

## 2. Results

### 2.1. Chemometric Analysis

#### 2.1.1. Hierarchical Cluster Analysis

**Figure 1:**
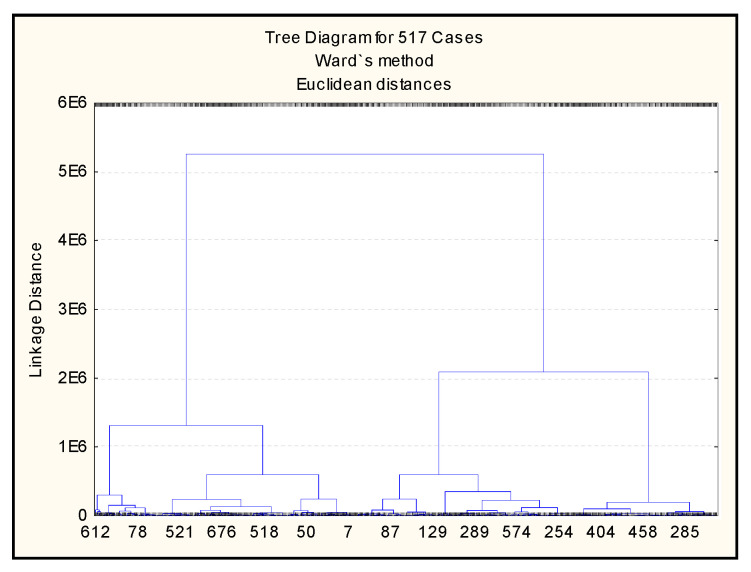
Dendrogram of hierarchical cluster analysis using Ward’s clustering method.

Since the compounds reported in ChEMBL come from heterogeneous sources, a preliminary analysis of the experimental data was performed to eliminate duplicate compounds and outliers, which could interfere with the machine learning process in the modeling. In this regard, a hierarchical cluster analysis was developed, which identified compounds linked at the zero level that had the same chemical structure but different response variables; those compounds were eliminated from the study. Figure 1 shows the dendrogram obtained with a Euclidean distance metric. No atypical behavior of the compounds was observed in the graph, although two groups that are structurally distinct are evident.

#### 2.1.2. Piecewise Regression Model

To develop the classification models, the continuous variable pIC_50_ was converted into a dichotomous discrete variable (either active or inactive class). This was achieved through piecewise regression analysis, which allows estimating two independent regression equations based on the breakpoint estimated by the program. The estimated equations, using a subset of predictor variables obtained through the genetic algorithm, are shown below:

y = 3.32 + 1.10 × **Mor26m** − 0.39 × **nR = Cs** − 0.37 × **C-008** − 0.38 × **B04[N-O]** − 0.11 × **F06[C-Cl]** − 0.06 × **F10[N-F]** (1)

y = 4.65 + 0.53 × **Mor26m** − 0.10 × **nR = Cs** − 0.18 × **C-008** − 0.19 × **B04[N-O]** + 0.03 × **F06[C-Cl]** − 0.04 × **F10[N-F]** (2)

R = 0.922            Explained variance = 84.977%            Breakpoint = 3.145

**Figure 2:**
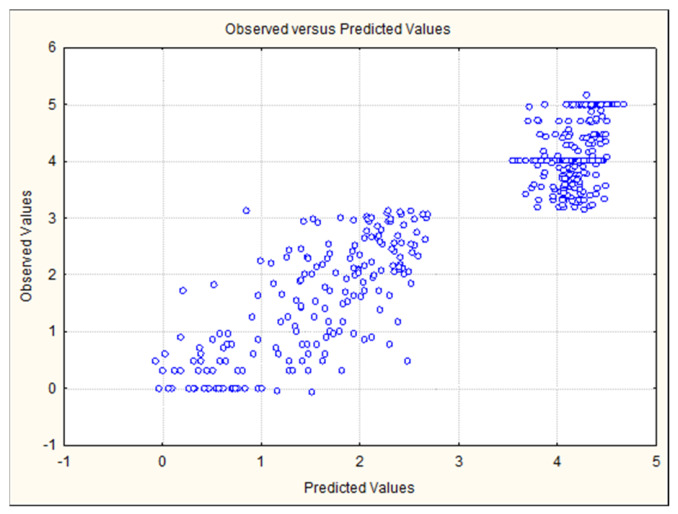
Observed values versus values predicted by piecewise regression.

In this study, this method is not used as a predictive technique, but rather as a tool for defining the classes. One equation is for y values ≤ 3.145 (Equation (1)) and another, for y values > 3.145 (Equation (2)). Figure 2 shows the observed values versus the values predicted by the piecewise model.

#### 2.1.3. k-Means Cluster Analysis

**Figure 3:**
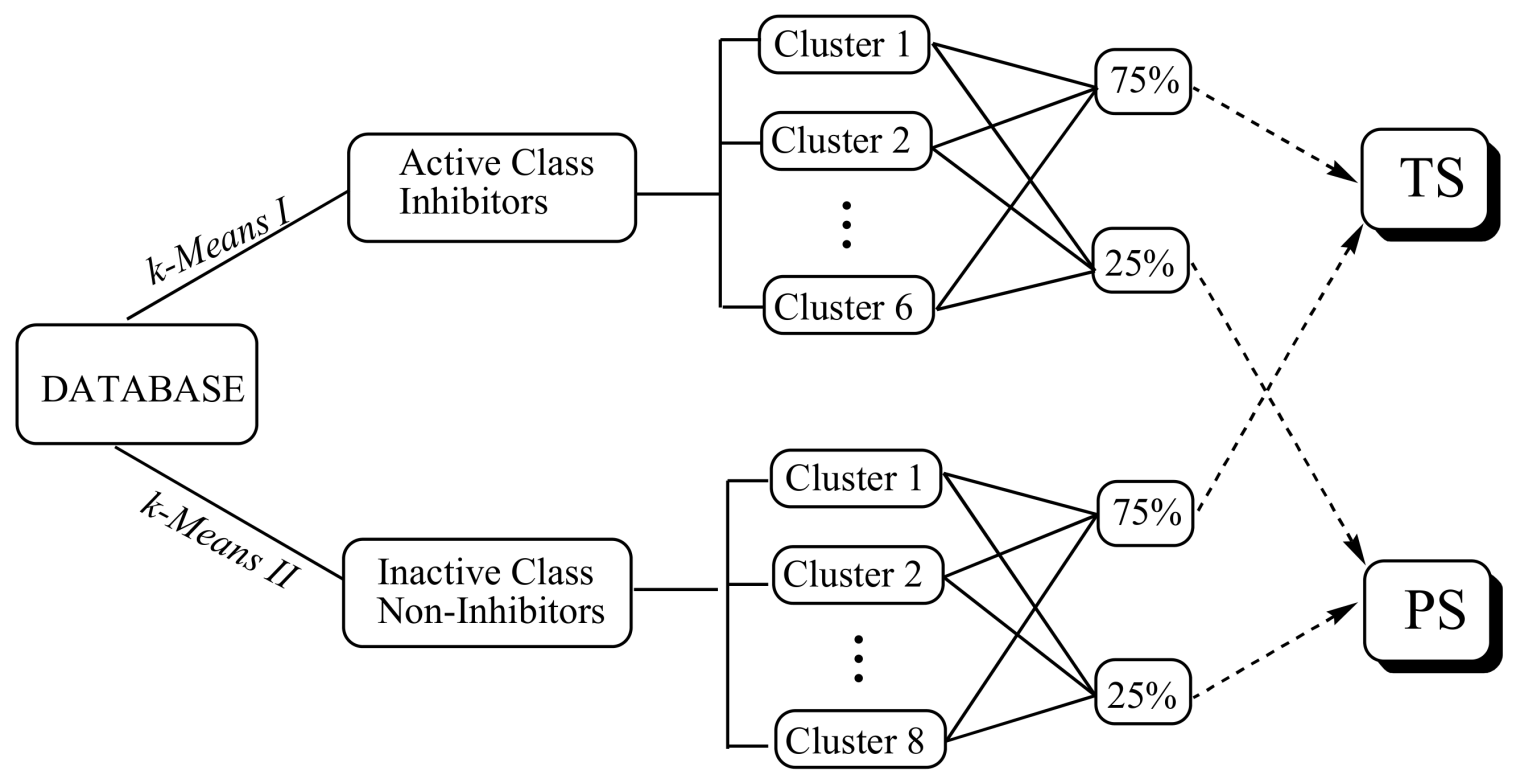
Selection of training and prediction series.

A *k*-means cluster analysis method was developed for each dataset to divide the database into training and prediction series, ensuring structural representativeness in both series. In this process, 25% of the compounds from each cluster were used for the external prediction set (PS), and the remainder constituted the training set (TS). Figure 3 shows the selection process for the training and prediction series.

#### 2.1.4. Linear Discriminant Analysis (LDA)

The following shows the equation of the general model obtained from the patterns generated by families of descriptors with their statistical parameters:

Class = − 4.24 + 5.64 × **BELe3** − 0.14 × **G(S..F)** − 2.23 × **Mor16m** − 1.96 × **nArCONH2** − 1.49 × **Mor20v** − 0.99 × **nArNHR** − 4.82 × **nN-N** − 2.19 × **C-008** − 1.86 × **N-071** (3)

N = 359            λ = 0.550            D^2^ =3.421            F = 31.563            *p* = 0.0001

C_TS_ = 0.649            Q_TS_ = 83.287            Sens_TS_ = 85.520            Spec_TS_ = 87.096            FPR_TS_ = 20.290

C_PS_ = 0.691            Q_PS_ = 85.000            Sens_PS_ = 87.143            Spec_PS_ = 87.143            FPR_PS_ = 18.000

where Class refers to the inhibitory activity of PLK-1, N is the number of compounds, λ is the Wilk’s statistic, D^2^ is the squared Mahalanobis distance, F is the Fisher ratio, *p* is the significance level, C is the Matthews correlation coefficient, Q is the accuracy of the model, Sens is the sensitivity, Spec is the specificity, and FPR is the false positive rate for the TS and PS.

#### 2.1.5. Artificial Neural Network Multilayer Perceptron (MLP)

**Figure 4:**
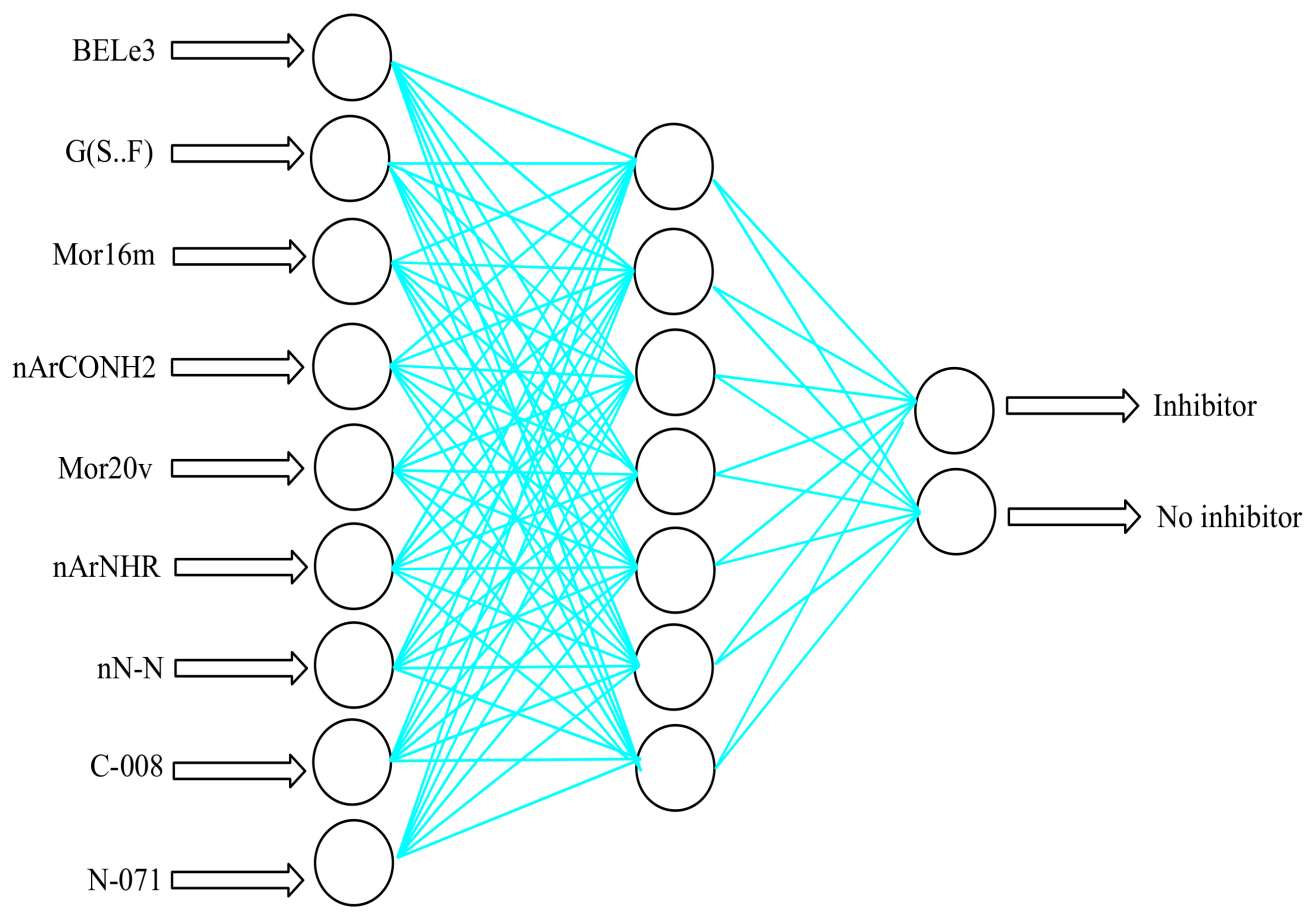
Schematic representation of the obtained MLP network architecture.

The MLP model features a 9-7-2 architecture, as shown in Figure 4. Furthermore, the training algorithm is the Broyden-Fletcher-Goldfarb-Shanno (BFGS) algorithm, with a sum of squares (SOS) error function, an exponential function for activating neurons in the hidden layer, and a hyperbolic tangent (Tanh) function for activating output neurons. Table 1 presents the statistical parameters used to evaluate the model’s performance in both the training and prediction series.

### 2.2. Applicability Domain (AD)

In this study, the AMBIT Discovery program was used to determine the AD of the classification models using Euclidean distance, Cityblock and Range methods. According to all three methods, all TS compounds are found within the experimental chemical space of the database. However, using the Range metric, four PS compounds were found to be out (compounds 159, 172, 343 and 443), of which compound 159 is also excluded by the Cityblock and Euclidean distance methods.

### 2.3. Virtual Screening

**Figure 5:**
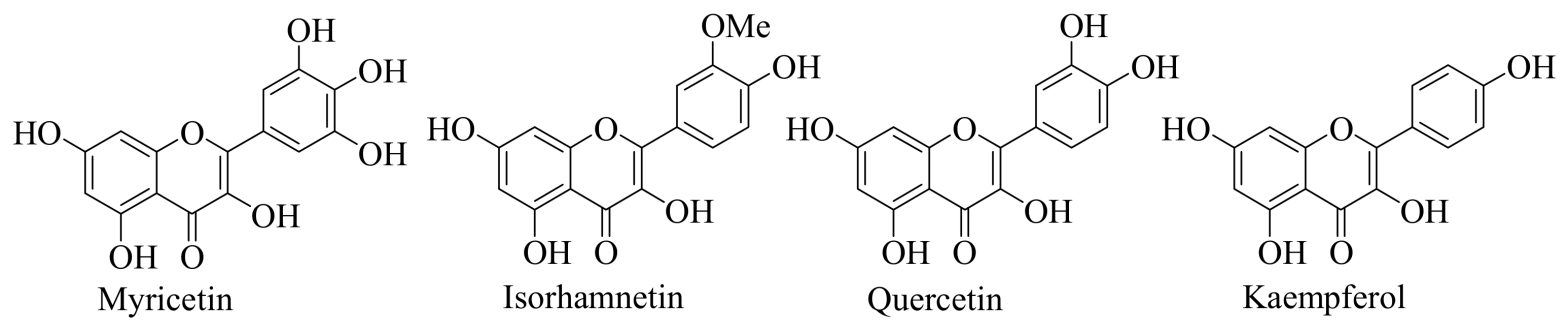
Chemical structure of the flavonoids identified in the virtual screening.

**Figure 6:**
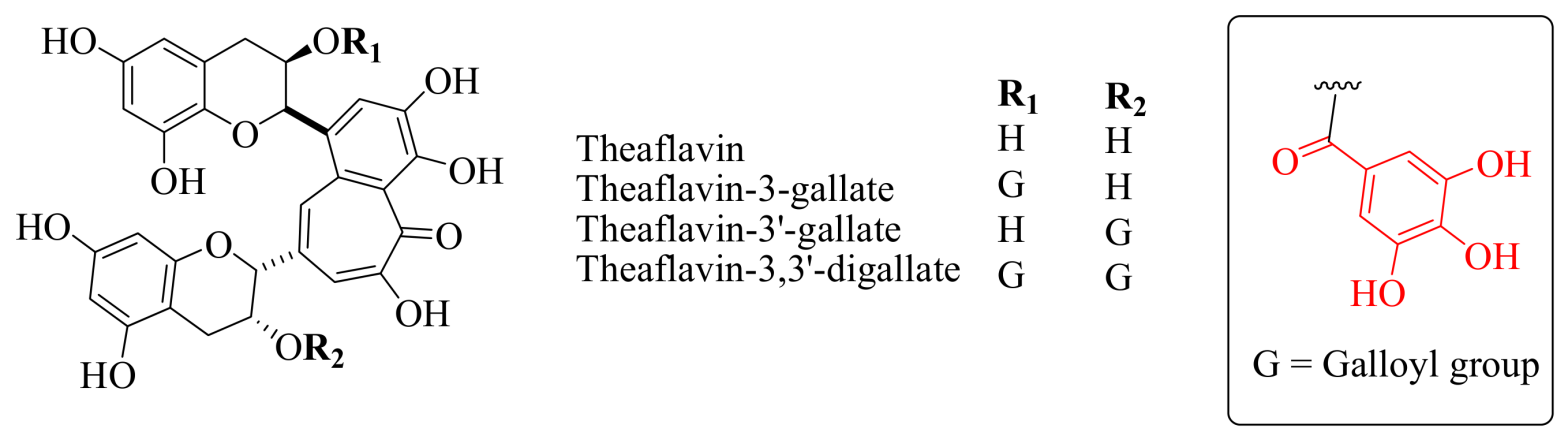
Chemical structure of compounds that do not meet oral bioavailability properties.

Table 2 shows the results of the analysis of 27 naturally occurring flavonoids, and Figure 5 shows the chemical structures of the most promising compounds as potential drug candidates. Four compounds (theaflavin and its three galloyl derivatives) do not meet the oral bioavailability properties shown in Figure 6.

### 2.4. Docking

**Figure 7:**
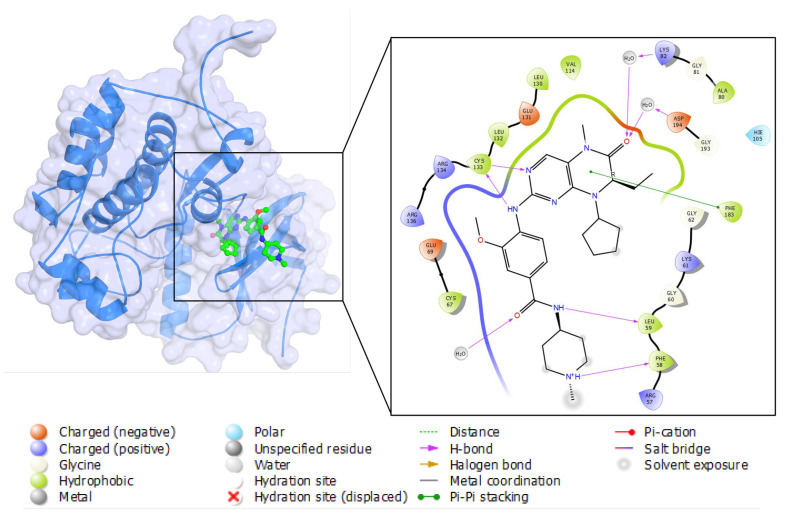
Protein–ligand interactions of BI-2536 at the active site of PLK1 (PDB: 2RKU).

The binding profile of dietary flavonoids to the catalytic domain of PLK-1 was investigated, using a hierarchical docking scheme in Glide [45,46]. This was implemented on the 2RKU crystallographic structure, using successively Standard Precision (SP) and Extra Precision (XP) algorithms, with thermodynamic scoring of the XP pose by Molecular Mechanics/Generalized Born Surface Area (MM/GBSA) on Prime [47]. The inhibitor BI-2536, co-crystallized in 2RKU, was used as a reference ligand to validate the protocol (Figure 7). The redocking yielded a heavy-atom RMSD of 1.13 Å, well below the established threshold (RMSD < 2.0 Å).

**Figure 8:**
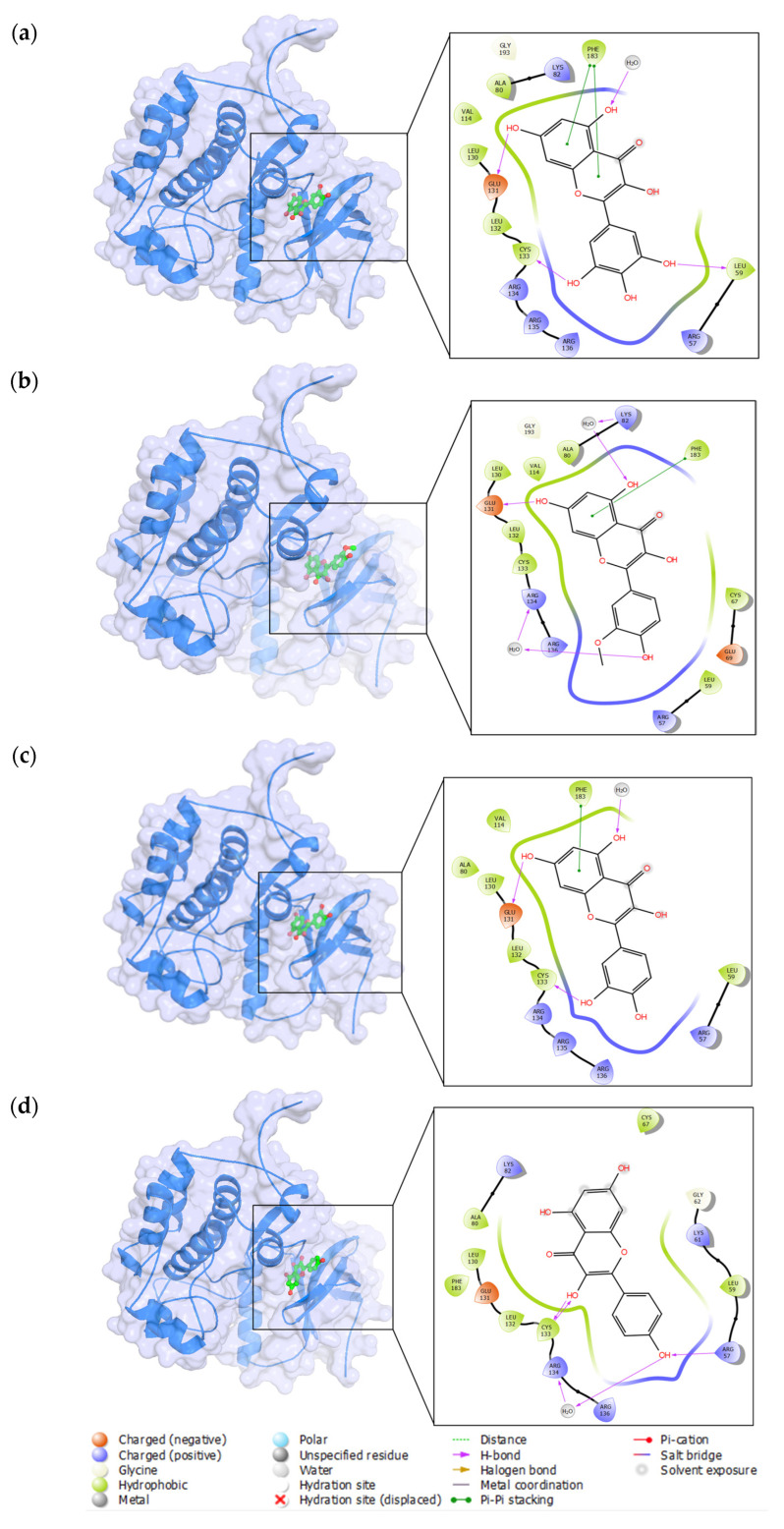
Interaction diagram of compounds in the catalytic site of PLK1: (**a**) myricetin, (**b**) isorhamnetin, (**c**) quercetin, and (**d**) kaempferol.

Table 3 summarizes the GlideScore SP and XP, along with the binding free energy (ΔG_bind_) derived from the MM/GBSA score applied to the best XP pose of each ligand, and Figure 8 illustrates the complex formed between PLK-1 and myricetin (a), isorhamnetin (b), quercetin (c), and kaempferol (d).

The SP and XP refer to Glide Standard Precision and Extra Precision scoring functions, respectively, expressed in Glide units. The ΔG_bind_ is the MM/GBSA binding free energy of the best XP pose under implicit solvation VSGB 2.0, in kcal·mol^−1^. For all three metrics, the more negative values denote thermodynamically more favorable protein–ligand binding, with magnitude proportional to the predicted affinity, consistent with ΔG = −RT ln Kd.

### 2.5. Molecular Dynamics

To complement the previous analyses, we conducted a single 300 ns molecular dynamics simulation as a preliminary evaluation of the stability and conformational behavior of the docked complexes.

#### 2.5.1. Exploratory Analysis of Complex Stability

**Figure 9:**
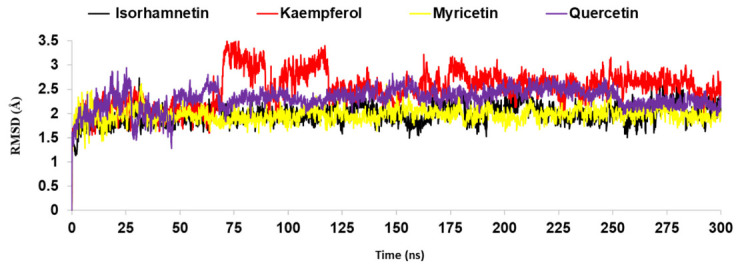
Time evolution of the RMSD of Cα atoms for 2RKU–flavonoid complexes.

The time evolution of the root mean square deviation (RMSD) of the Cα atoms for the four 2RKU–flavonoid complexes, over 300 ns of molecular dynamics simulation, are shown in Figure 9. In each plot, the RMSD data (in Å) were calculated with respect to the initial minimized structure. The RMSD values of Cα, in the 200–300 ns equilibrium window, for each complex are shown in Table 4. The values represent the mean ± standard deviation calculated over 3000 conformations per complex (sampling every 100 ps), extracted from the entire path. The RMSD is calculated on the Cα complexes relative to the initial minimized structure. The complexes are presented in order of increasing average RMSD.

#### 2.5.2. Residue-Specific Flexibility

**Figure 10:**
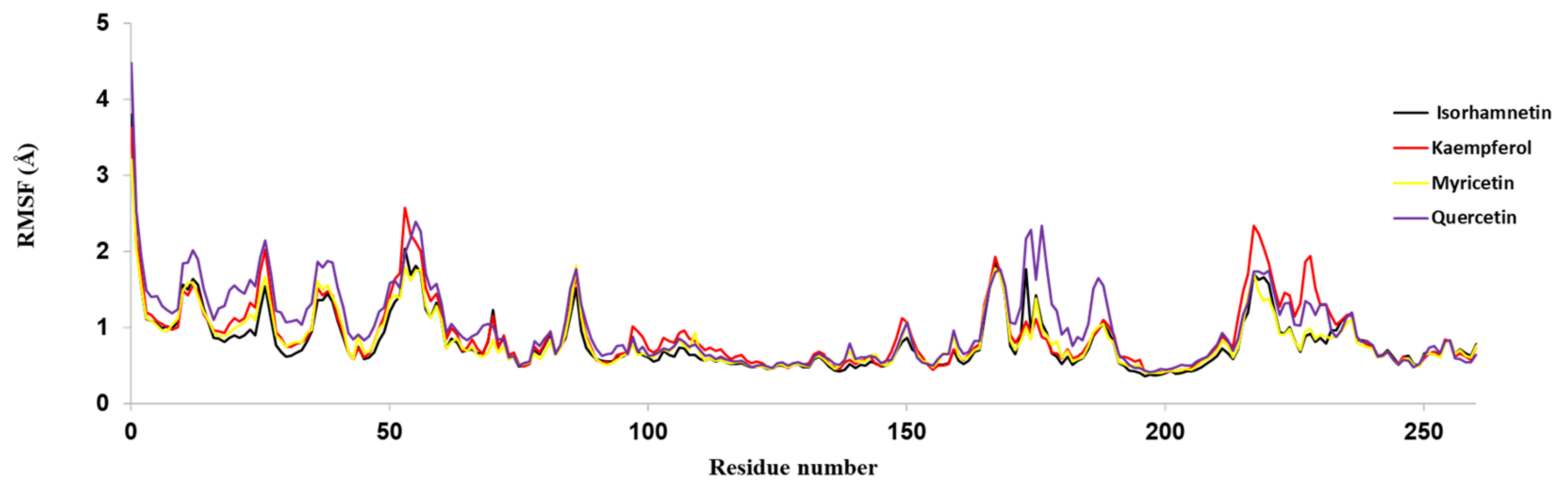
RMSF of residue-specific Cα atoms in 2RKU-flavonoid complexes.

The root mean square fluctuation (RMSF) profile of 2RKU-flavonoid complexes along the polypeptide chain is shown in Figure 10. The *x*-axis shows the crystallographic numbering of the PLK-1 kinase domain (residues 37–330 of chain A of Protein Data Bank (PDB) identifier 2RKU). The shaded bands identify the functional regions of interest: P-loop (residues 53–63), hinge (131–136), activation loop (194–220), and αF–αG loop (253–260). The mapping between the output file index and the crystallographic numbering is established as index + 37 = crystallographic residue. Table 5 shows the overall magnitude and RMSF values of key ATP-binding site residues of the four 2RKU–flavonoid complexes along a 300 ns path.

#### 2.5.3. Structural Compactness Analysis

**Figure 11:**
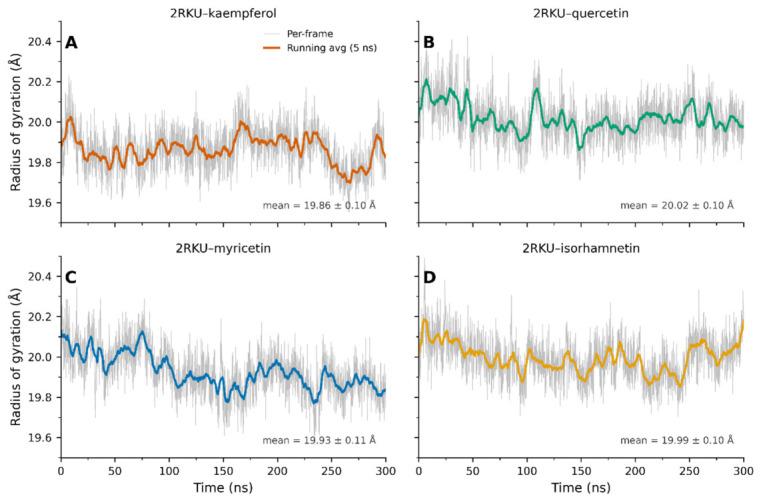
Time evolution of the radius of gyration of the PLK-1 kinase domain during 300 ns of molecular dynamics simulation; the per-frame trace (grey) is overlaid with its 5 ns running average (colored line). (**A**) 2RKU–kaempferol, (**B**) 2RKU–quercetin, (**C**) 2RKU–myricetin, and (**D**) 2RKU–isorhamnetin.

The time evolution of the radius of gyration (RG) of the PLK-1 kinase domain over 300 ns of molecular dynamics simulation, for the four 2RKU–flavonoid complexes, is shown in Figure 11. The ordinate axis is kept within a narrow range of 1.0 Å (19.5–20.5 Å) to allow visual reading of the fluctuations, since a wider range would mask the differences between complexes. The values of the magnitudes along the full path (0–300 ns) and in the equilibrium window (200–300 ns) can be seen in Table 6. These magnitudes are derived from 3000 conformations per path (sampling every 100 ps). The “RG Range” indicator shows the minimum and maximum values explored during the full 300 ns. The complexes are arranged in increasing order of mean RG, and the correspondence between the global mean RG and the mean plateau RG is consistent with the dimensional convergence of the folding. A 5 ns running average is overlaid on the per-frame trace to aid visual interpretation of the trend.

#### 2.5.4. Free Energy Landscape Topography

**Figure 12:**
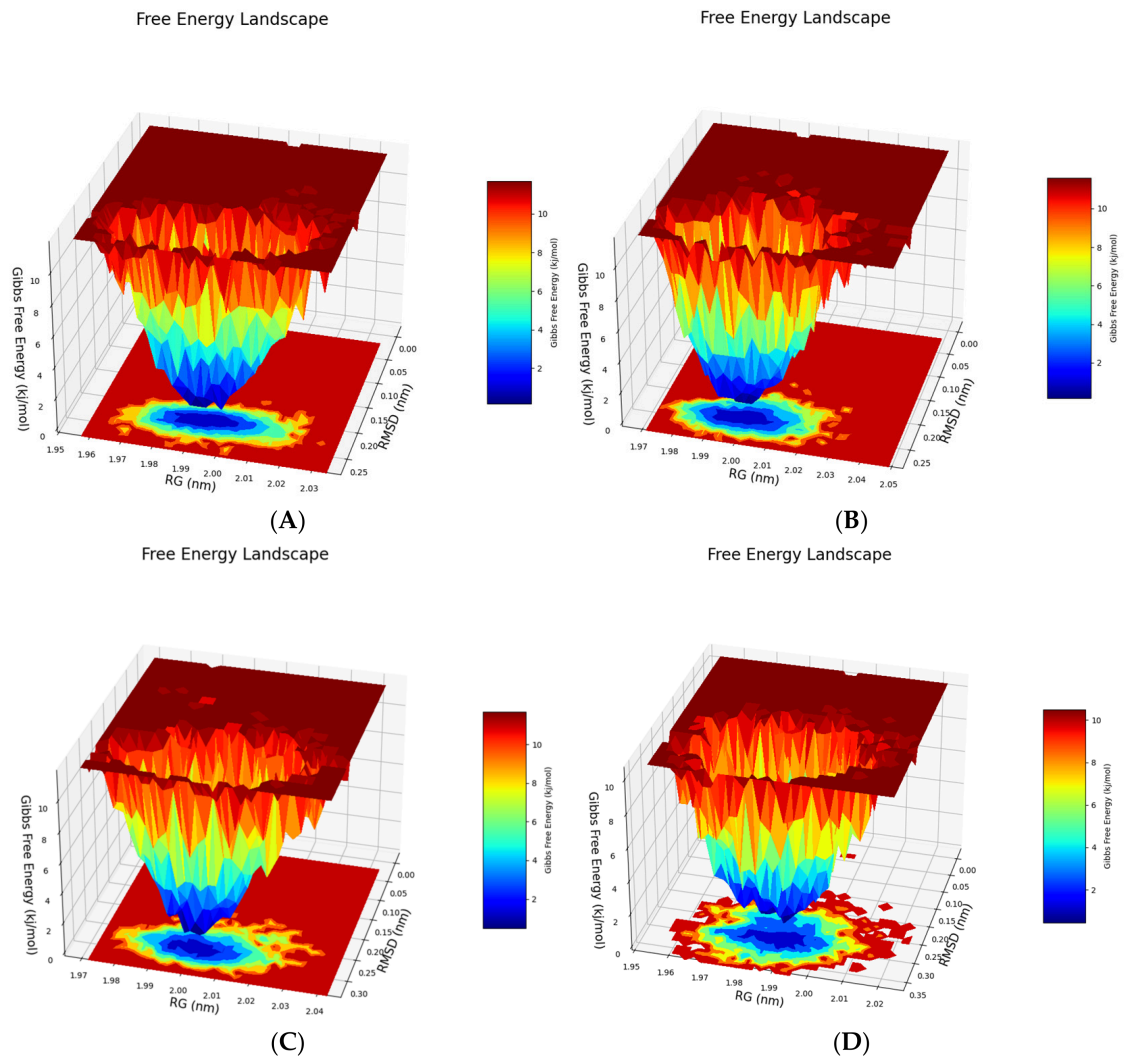
Three-dimensional free energy landscape of the conformational ensemble for the complexes. (**A**) 2RKU–myricetin, (**B**) 2RKU–isorhamnetin, (**C**) 2RKU–quercetin, and (**D**) 2RKU–kaempferol.

The reconstruction of the free energy landscape (FEL), in the reactive coordinate (RMSD, RG), projects the probability distribution of the conformational ensemble onto an energy surface, which allows the identification of sampled low-energy conformational states and the quantification of the relative depth of associated basins. The resulting topography for the four complexes is shown in Figure 12, and the extracted quantitative parameters are summarized in Table 7. Gibbs free energy (ΔG, in kJ/mol) is color-coded according to the scale shown in the common sidebar, with ΔG = 0 kJ/mol assigned to the global minimum of each landscape. The horizontal axis shows the combined coordinate derived from the structural RMSD (in units of the rescaled grid used by the FEL protocol); the vertical axis shows the radius of gyration (in nm). The surface is obtained from a grid of 16,896 points per complex. The parameters are derived from the two-dimensional FEL grid of 16,896 points reconstructed from the 3000 frames of each trajectory, using the protocol implemented in G_Measures v.0.9d. The global minimum is operationally set to ΔG = 0 kJ/mol in each landscape. The horizontal coordinate of the minimum corresponds to the combined/rescaled RMSD coordinate as defined by the FEL protocol and is expressed in arbitrary grid units (a.u.), not in the RMSD structural units of the set. The vertical coordinate is reported in native RG units (nm). The number of points within each energy threshold quantifies the relative extent of the low-energy basins. Across the four complexes the landscape collapses into a single global minimum with no competing basins, indicating a single, well-defined bound macrostate; the radius-of-gyration coordinate of this minimum (~19.9 Å) matches the equilibrium RG, and the comparative depth and breadth of the minima (Table 7) quantify the differential conformational confinement among the flavonoids.

#### 2.5.5. Protein–Ligand Interaction Footprint

Geometric characterization of protein–ligand contacts maintained throughout the 300 ns allows identification of ATP binding-site residues, supporting molecular recognition beyond the static docking state.

**Figure 13:**
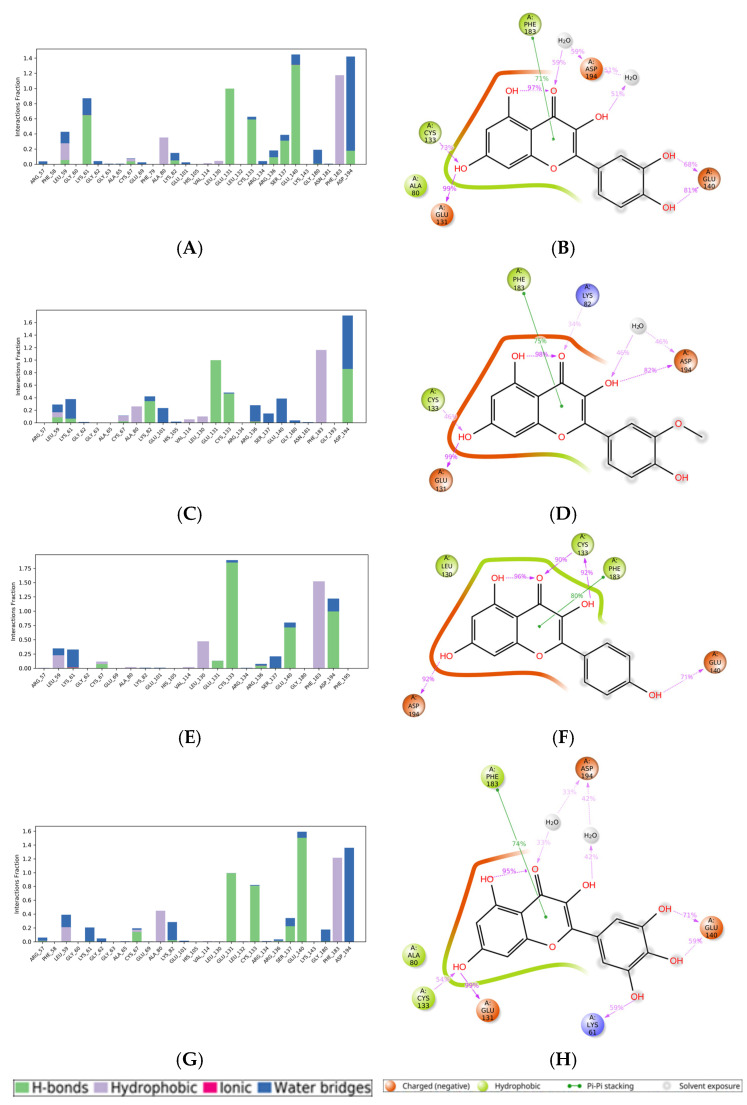
Protein–ligand interaction fingerprint for the four 2RKU-flavonoid complexes, obtained by persistent contact analysis along a 300 ns path. (**A**,**B**) 2RKU-myricetin; (**C**,**D**) 2RKU-isorhamnetin; (**E**,**F**) 2RKU-kaempferol; (**G**,**H**) 2RKU-quercetin.

Figure 13 integrates two complementary levels of analysis for each of the four complexes: the residue occupancy histogram broken down by interaction type (Figure 13A,C,E,G), which quantifies the cumulative presence of each residue during the trajectory, and the two-dimensional active site diagram (Figure 13B,D,F,H), which maps atom by atom the specific contacts maintained above 30% of the frames. The values in the two-dimensional diagram correspond to specific atomic contacts and, by design, are equal to or less the total residue occupancy shown in Table 8, according to the data generated by the SID module. Only residues with an occupancy ≥ 5% in at least one of the four complexes during the 300 ns path are shown.

Occupancy rates, expressed as percentages, were calculated as the ratio between the number of unique frames in which the contact was present and the total of 3001 frames sampled per path. A dash indicates <5% occupancy or the absence of a contact in the corresponding complex. The residue numbering corresponds to the 2RKU crystallographic structure (A chain). Ionic, metal coordination, and π-cationic contacts are not included in the table because they do not exceed the relevant persistence threshold in any of the four complexes.

#### 2.5.6. Thermodynamic Affinity Analysis by MM-GBSA

Binding-free energy decomposition analyses were performed using the MM/GBSA protocol with implicit solvation VSGB 2.0. The mean values of ΔG_Bind_ and its unbound components are summarized in Table 9.

The values represent the mean ± standard deviation calculated over 3000 equidistant frames of the 300 ns production path. All quantities are expressed in kcal·mol^−1^. In addition, ΔG_Bind_ includes the packing contributions (ΔG_Packing_ ≈ −3.2 to −4.3 kcal·mol^−1^) and the covalent penalty associated with geometric strain (ΔG_Covalent_ ≈ +1.6 to +3.0 kcal·mol^−1^). The contribution of the nonpolar surface area (ΔG_Solv-SA_) was not computed separately in this VSGB 2.0 configuration and is absorbed within the ΔG_Lipo_ term. Negative values of ΔG_Bind_, ΔG_Coulomb_, ΔG_HBond_, ΔG_Lipo_, and ΔG_vdW_ denote energetically favorable contributions to binding, whereas the positive value of ΔG_Solv-GB_ corresponds to the polar desolvation penalty.

The convergence, temporal consistency and statistical robustness of the single-trajectory essential-dynamics analyses, together with the multi-method clustering and the inter-ligand comparison on a common essential subspace, are provided in the Supplementary Materials (Figures S1–S3, Tables S1–S5). Because a single 300 ns production trajectory may undersample the slowest collective modes, these analyses are used to assess the robustness of the sampled conformational behavior and to support the observed structural stability and conservation of the binding mode, rather than to infer quantitative state populations or exhaustive conformational sampling. The MM-GBSA results are therefore interpreted as comparative energetic estimates rather than as evidence of a definitive affinity ranking.

## 3. Discussion

In this study, a computational approach combining ligand-based and structure-based methods was used to identify potential antitumor drug candidates. First, chemometric tools were used to extract information from a database of PLK-1 inhibitor compounds to train classification models, useful for discriminating between inhibitor and non-inhibitor compounds.

Piecewise regression analysis was not used as a predictive technique, but rather as a tool to convert the continuous response variable into a discrete variable and to divide the dataset into two groups or classes. The graph of observed values *versus* predicted values shows the presence of two separable groups of compounds (Figure 2). Therefore, based on this study, the case base was divided into two classes, using the breakpoint as a reference. It is important to note that all compounds have an experimental *p*IC_50_ value, so within the class of inactive compounds, there could be some that show, to some extent, PLK-1 inhibitory activity. However, it is not necessary to identify new compounds with this activity, as the goal is to find new candidates with potent activity (strong inhibitors).

Using the discrete variable obtained, a qualitative LDA study was performed. This is an important tool in computational chemistry due to the simplicity of the method and its widespread use in the literature for predicting chemical and pharmacological properties, which is why it was chosen for this study. This model correctly classified over 80% of the training set using only nine predictor variables, adhering to the principle of maximum parsimony. Furthermore, the Matthews correlation coefficient (C = 0.649) quantifies the strength of the linear relationship between the molecular descriptors and the classifications. Along with precision, other parameters such as sensitivity, specificity, and false positive rate are commonly used in medical statistics and are suitable for the model. However, the most important criterion for accepting or rejecting the discriminant model is based on the statistics for the external prediction set, known as the model’s predictive power. The prediction set showed statistical parameters slightly superior to those of the training set (C = 0.691, Q = 85.000, Sens = 87.143, Spec = 87.143, and FPR = 18.000).

The MLP model, which uses artificial neural networks (ANNs) to classify PLK-1 inhibitors and non-inhibitors, is based on modeling the brain’s learning capacity, making it an increasingly common tool in classification tasks. The overall goal is to assign the appropriate class to each case based on the network’s prior learning. As can be seen, this neural network achieves excellent overall classification, sensitivity, specificity, and Matthews correlation coefficient values, while reducing the false positive rate for both the training and prediction series. Furthermore, the false positive rate is less than 20 for both sets, a crucial parameter for predicting new compounds with PLK-1 inhibitory activity. The area under the receiver operating characteristic (ROC) curve was also calculated, obtaining results close to one (0.923 for the training set and 0.933 for the prediction set), which confirms the strong discriminatory power between classes.

The AMBIT Discovery program was used to determine the model’s AD. This is an open-source, user-friendly application that estimates AD in QSAR models, by evaluating structural similarity in descriptor space with a set of chemical compounds as a function of distance. In this study, compound 159 can be considered an outlier and was excluded from the database because its predictions are unreliable.

Considering the quality of the obtained models, a virtual screening was performed combining the LDA and MLP outputs to estimate the class to which the compounds belong. Four compounds containing a theaflavin core were identified that do not meet the oral bioavailability criteria. This is due to their molecular weight being greater than 500 Da, as well as their highly polar nature and large number of hydroxyl groups, which can act as hydrogen-bond donors or acceptors. Recent studies indicate that these compounds are not absorbed in significant quantities in the upper gastrointestinal tract. This malabsorption is attributed to factors such as their molecular structure, pH-dependent instability, and interaction with cellular transport mechanisms [47,48]. Furthermore, the disease under study is a chronic condition, and the choice of one route of administration over another affects the patient’s quality of life; therefore, these compounds are excluded from the study. All compounds fall within the model’s AD, except for the theaflavin compounds, indicating that the predictions are reliable, except in these cases. Of the total number of compounds, 15 were identified as active compounds by both models, and of these, isorhamnetin, kaempferol, myricetin, and quercetin show a high probability of belonging to the active class. These compounds fulfill Lipinski’s rule of five. These flavonoids are considered promising drug candidates, suggesting that consuming flavonoid-rich foods may be beneficial health effects, associated with lower toxicity and a better pharmacokinetic profile, helping to prevent or inhibit the development of diseases such as cancer [49].

To complement the results obtained with the ligand-based mathematical model, the binding energy to the enzyme’s active site, as well as its partitional analysis, were also considered through molecular docking. The autodocking of BI-2536 recovered the co-crystallized type-I bonding mode (Figure 7), with a bidentate anchor on the hinge region: a directional H-bond with the carbonyl of the Cys133 main chain (d = 2.084 Å; H-bond = −1.382 kcal/mol; Eint = −6.356 kcal/mol) and a salt bridge with Glu131 (d = 2.455 Å; Coul = −10.531 kcal/mol; Eint = −11.632 kcal/mol). The pteridinone core occupied the adenine pocket flanked by Leu59 (Eint = −13.841 kcal/mol; VdW = −9.986 kcal/mol), Leu132, Val114, and Phe183, through a dense hydrophobic enclosure consistent with the distinctive feature reported for type-I kinase inhibitors [50]. Two crystallographic water molecules (HOH_563 and HOH_527; H-bond ≈ −1.000 kcal/mol each) were retained in the docked pose, completing the polar network observed in the crystallography. The geometric and energetic fidelity of the re-docking validates the receptor grid and scoring parameters and provides the upper limit of affinity, achievable under the applied protocol [51,52].

Myricetin was positioned as the highest-ranked flavonoid in the series (SP = −10.403; XP = −10.444; = −51.68 kcal/mol). Its chromone core reproduced the bidentate hinge anchoring exhibited by the reference ligand, with simultaneous H-bonds towards Glu131 (d = 1.762 Å; H-bond = −0.972; Eint = −6.231 kcal/mol) and Cys133 (d = 1.944 Å; H-bond = −1.000; Eint = −6.198 kcal/mol). The aromatic C–A system coupled to the adenine pocket via VdW contacts with Leu59 (Eint = −8.827 kcal/mol; VdW = −5.433 kcal/mol) and a face-to-face π-stacking interaction with Phe183 (VdW = −5.214 kcal/mol; Figure 8a). The pyrogallol-B (3’,4’,5’-OH) motif projected multiple hydrogen donors toward the leading edge of the ATP pocket, where it recruited cation-π interactions with Lys82 (Coul = −2.203 kcal/mol) and a salt bridge with Arg57 (Coul = −4.773 kcal/mol). Two bridging water molecules (HOH_698 and HOH_563) coupled to the polar network as weak but stable conformational mediators. This distinctive feature of the polyfunctional binding, characteristic of highly hydroxylated flavonols toward kinases, justifies the higher calculated affinity of the series and is consistent with the SAR consensus for this scaffold family [53,54,55].

Quercetin reproduced the bidentate hinge anchoring (SP = −9.741; XP = −9.777; ΔG_bind_ = −45.34 kcal/mol), with a H-bond toward Glu131 at 1.817 Å (H-bond = −0.998; Eint = −7.119 kcal/mol) and a H-bond toward Cys133 at 1.853 Å (H-bond = −1.000; Eint = −6.567 kcal/mol). The preservation of the double-hairpin hinge coupling, along with the most intense π-stacking in the entire series at Phe183 (VdW = −5.454 kcal/mol; Figure 8c), shows that catechol-B (3’,4’-dihydroxy) confers the most complementary axial geometry to the adenine pocket. Hydrophobic packing with Leu59 (Eint = −5.951 kcal/mol), a π-cation with Lys82 (Coul = −3.930 kcal/mol), and a water-mediated bridge on HOH_698 (H-bond = −0.988 kcal/mol) complete the polar network. This distinctive feature of the binding is consistent with the experimentally reported submicromolar inhibitory activity of quercetin, against other related cancer kinases [56].

Isorhamnetin showed the lowest GlideScore in the series (SP = −8.969; XP = −9.005), but the ΔG_bind_ recovered by MM/GBSA (−45.93 kcal/mol) positioned it marginally above quercetin, establishing a rank inversion between the empirical score and the thermodynamic re-scoring, thus allowing for direct structural rationalization. The 3’-OMe methylation of ring B abolished the H-bond toward Cys133; where the ligand was located at 1.990 Å but without an acceptable directional geometry (Eint = −2.904 kcal/mol without H-bond), leaving the H-bond with Glu131 as the only hinge anchor, formed at the shortest distance in the entire series (1.662 Å; H-bond = −1.000; Eint = −6.028 kcal/mol). The single-ended hinge coupling was preserved by an expanded leading-edge electrostatic network: a π-bonded cation reinforced with Lys82 (Coul = −4.184 kcal/mol) and a unique salt bridge with Glu69 (Coul = −4.192 kcal/mol), absent in the other congeners, both linked by hydroxyls groups on ring B and ring A. Edge-to-face π-stacking with Phe183 (VdW = −5.000 kcal/mol) and packing with Leu59 (Eint = −7.195 kcal/mol; Figure 8b) preserved hydrophobic complementarity. In particular, the water-mediated bridging in HOH_698 reached the highest magnitude among the hydroxylated flavonoids (H-bond = −1.000; Eint = −3.454 kcal/mol). The inability of the empirical Glide function to capture adequately distributed electrostatic contributions and non-additive solvation terms is well documented for systems with extensive polar lattices, and it is precisely the type of discrepancy that the MM/GBSA re-evaluation is designed to resolve [57,58].

Kaempferol obtained the second-best GlideScore in the series (SP = −10.193; XP = −10.229), above quercetin and isorhamnetin, but the MM/GBSA re-evaluation relegated it to last place in the thermodynamic ranking (ΔG_bind_ = −41.11 kcal/mol). The discrepancy between the docking and MM/GBSA finds an immediate explanation in the residual pose analysis: The ligand formed an exceptionally strong H-bond with Cys133 (d = 2.073 Å; H-bond = −1.777; Eint = −8.349 kcal/mol; the strongest in the series), but contact with Glu131 collapsed to an electrostatically repulsive regime (Coul = +2.719 kcal/mol; Eint = +1.981 kcal/mol), abolishing the second arm of the bidentate hinge coupling. The absence of the 3’-OH group shifted ring B from optimal geometric complementarity, weakening the π-stacking at Phe183 (VdW = −3.866 kcal/mol; the lowest in the series; Figure 8d). Residual packing at Leu59 (Eint = −6.551 kcal/mol) and a secondary polar H-bond with Cys67 (H-bond = −0.295 kcal/mol) did not compensate for the loss of the second arm. The effective affinity was partially rescued by a water-mediated bridge on HOH_698 with remarkably strong values (d = 1.750 Å; H-bond = −4.471; Eint = −4.042 kcal/mol), a pattern consistent with the well-characterized role of structural water molecules as conformational mediators in kinase-inhibitor complexes with incomplete polar networks [59].

The calculated affinity hierarchy, BI-2536 ≫ myricetin > isorhamnetin ≈ quercetin > kaempferol, follows the B-ring substitution and hydroxylation pattern of ring B and, more specifically, how each substitution pattern modulates the kinase’s polar recognition profile. The pyrogallol moiety of myricetin (3′,4′,5′-triOH) exhibited the highest affinity (ΔG_bind_ = −51.68 kcal/mol) by maximizing hydrogen-bond interactions with residues at the pocket entrance, including Glu140, a dual contact with Lys61, and a bidentate hinge-anchoring interaction involving Glu131 and Cys133. The progressive elimination of hydroxyl groups in quercetin (3′,4′-diOH) and kaempferol (4′-OH) weakens this interaction network, which explains the lower affinity observed for kaempferol (−41.11 kcal/mol). Although kaempferol forms the strongest hydrogen bond with Cys133 among the series, the loss of the second hinge interaction and the absence of compensatory peripheral polar contacts prevent this contribution from translating into a higher overall affinity. Isorhamnetin (3′-OMe/4′-OH, −45.93 kcal/mol) represents the most informative case from a mechanistic point of view. Methylation of the 3′-hydroxyl group eliminates the direct hydrogen bond with Cys133, but does not reduce the relative affinity for quercetin. This loss is compensated for by a reorganization of electrostatic interactions, characterized by an optimized hydrogen bond with Glu131, an enhanced cation–π interaction with Lys82, and a unique salt bridge with Glu69, complemented by a water-mediated network connecting with Asp194 within the DFG motif. Consequently, the 3′-methoxy group behaves as a functionally equivalent substitute for the 3′-OH group in PLK-1, replacing a direct interaction with a combination of charge-assisted and water-mediated contacts of comparable energetic contribution. This behavior is consistent with the established SAR of bioactive flavonols, in which B-ring hydroxyl groups promote polar recognition at the entrance to the adenine-binding pocket, while water bridges and salt bridges provide compensatory interaction pathways that mitigate the energetic impact of the loss of individual direct contacts [53,55].

The binding mode adopted by the flavonoids differs qualitatively from that exhibited by BI-2536, in terms of the spatial distribution of charge on the active site. The reference inhibitor anchors its pteridinone core via a deep hydrophobic backbone (Leu59 with Eint = −13,841 kcal/mol; complemented by Leu132, Val114, and Phe183) and a single salt bridge with Glu131 (Coul = −10,531 kcal/mol), a distinctive feature of the type-I lipophilic mode of optimized synthetic inhibitors [51]. Flavonoids, on the other hand, distribute polar binding across multiple charged leading-edge residues: Arg57 (myricetin), Lys82 (all four), Glu69 (isorhamnetin), and Arg134/Arg136 (visible as secondary contacts in the 2D diagrams of Figure 8). This organization is consistent with a qualitatively distinct polyphenolic binding mode, consistent with the systematic analysis by Lešnik et al. [59] on polyphenol-protein interactions using the PDB database. This multi-residue charge distribution may confer selective promiscuity favorable to flavonoids in oncological contexts, where moderate and polyathergic inhibition with a reduced toxicity profile in prolonged use is sought. Detailed Structure-Based Analysis of Flavonoid–PLK-1 Complexes can be found in Section S1 of Supporting Information.

Although molecular docking provides valuable information about the putative binding mode of ligands, it only explores a limited region of the conformational landscape and relies primarily on a rigid receptor approximation. Furthermore, MM/GBSA binding free energies were obtained using an implicit solvent model, which ignores the explicit contribution of solvent molecules and only partially considers entropic effects. Therefore, to assess the dynamic stability of the predicted complexes, to verify the persistence of key protein–ligand interactions, and to capture conformational fluctuations inaccessible to docking calculations, 300 ns molecular dynamics simulations were performed.

When evaluating the global stability of the complexes in the molecular dynamics simulation, the four trajectories converged within the first 100 ns and remained plateaued until the end of the simulation (Figure 9). Furthermore, in the 200–300 ns equilibrium window, the RMSD of Cα was ordered as myricetin (1.996 ± 0.125 Å) < isorhamnetin (2.052 ± 0.197 Å) < quercetin (2.352 ± 0.179 Å) < kaempferol (2.587 ± 0.178 Å) (Table 3). This trend is qualitatively consistent with the QSAR predictions. In this study, no complex exceeded 3.2 Å, a range consistent with preservation of the overall kinase-domain fold throughout the simulation and with no evidence of global rearrangements or partial unfolding. The 2RKU-kaempferol system was the only one that displayed a second conformational adjustment after the initial equilibration, where the mean RMSD increased from 2.047 Å in the first 50 ns to 2.576 Å between 50 and 100 ns before stabilizing. The other three systems progressively relaxed from initial values of 1.8–2.1 Å to their respective plateaus without discontinuities. The dispersion in the equilibrium window modulates this rearrangement. Myricetin (σ = 0.125 Å) samples the narrowest part of the set; isorhamnetin, quercetin, and kaempferol (σ ≈ 0.18–0.20 Å) explore slightly wider, yet well-defined equilibrium fluctuations.

According to residue-specific flexibility analysis of the studied complexes, the RMSF profile along the polypeptide chain (Figure 10) describes a rigid core of the kinase domain, interrupted by localized fluctuations in a limited set of peripheral loops. The fraction of residues with RMSF < 1.0 Å reached 75.9% in both myricetin and isorhamnetin, decreased to 65.3% in kaempferol, and fell to 57.1% in quercetin (Table 4), reproducing the overall stiffness order identified in Section 2.5.1. The ATP-site residues involved in competitive ATP recognition remained largely rigid over the 300 ns in the four complexes. The hinge region (Glu131, Leu132, Cys133) showed RMSF values between 0.58 and 0.77 Å, Phe183 did not exceed 0.57 Å in any system, and Lys82 ranged from 0.59 to 0.84 Å. This invariance in the canonical determinants of competitive ATP docking supports the dynamic persistence of protein–ligand interactions, consistent with the binding mode proposed by docking. The most pronounced fluctuations are concentrated in three regions, as shown in Figure 10. The N-terminal (Ala37–Lys38) and C-terminal (Ile327–Ser330) ends show the highest values in the set (up to 8.8 Å at the C-terminus of quercetin), expected behavior in chain-end residues, and with no mechanistic implications for recognition. The αC–β4 loop, centered on Leu90–Pro92–His93, emerges as a flexible region in all four complexes, with RMSF values scaling from 1.74 Å (Pro92 in myricetin) to 2.57 Å (Leu90 in kaempferol). The reproducibility of the peak in all four systems suggests that the intrinsic flexibility of the segment that connects the αC-helix with the β4-sheet of the N-lobe remains flexible despite ligand binding. Two additional regions introduce ligand-dependent differences. In the glycine-rich P-loop, the quercetin complex raises Arg57, Leu59, and Lys61 to 1.55, 1.45, and 1.54 Å, respectively, compared to uniformly lower values of 1.27 Å in the other three systems. The P-loop covers the roof of the ATP site, and its greater local mobility in quercetin is consistent with a local reorganization of the active site aligned with the greater RMSD drift observed in this complex. The Val210–Gly213 segment of the activation loop, immediately C-terminal to the DFG motif (Asp194–Phe195–Gly196), registers RMSF values of 2.17 to 2.33 Å in quercetin compared to <1.77 Å for the other three complexes; its mobilization suggests a coupling between the binding mode of this flavonoid and the dynamics of the regulatory zone of the catalytic cycle. Kaempferol exhibits a distinctive pattern, located in the αF–αG-loop of the C lobe. Ser254, Cys255, and Leu256 register RMSF values of 2.34, 2.23, and 2.05 Å, respectively, between 0.4 and 0.6 Å higher than the equivalent values in the other three complexes. This region does not participate directly in ligand recognition, so its flexibility suggests dynamic propagation from the ATP binding site toward the distal surface of the C lobe.

According to structural compactness analysis, the RG of the kinase domain ranged from 19.86 Å (kaempferol) to 20.02 Å (quercetin) over 300 ns, with σ ≤ 0.12 Å in all four complexes (Figure 11A–D, Table 6). The 0.16 Å difference between the most compact and most expanded systems is consistent with the series remaining within a single globular folding state, with no evidence of interlobular opening or progressive relaxation of the hydrophobic core. The maximum RG explored for each pathway (0.79 Å (myricetin), 0.78 Å (isorhamnetin), 0.72 Å (quercetin), and 0.68 Å (kaempferol)) is consistent with thermal fluctuations superimposed on a single structural minimum. Furthermore, the time series do not record transitions to partially unfolded conformations. Quercetin exhibits the highest mean RG of the set (20.02 ± 0.10 Å), 0.16 Å higher than kaempferol (19.86 ± 0.10 Å). This modest increase is qualitatively consistent with the greater flexibility observed in the P-loop and activation loop in the RMSF analysis. Myricetin (19.93 ± 0.11 Å) and isorhamnetin (19.99 ± 0.10 Å) occupy intermediate positions. The four systems appear to preserve native compactness over the simulated interval, with variations between ligands below the threshold of low-resolution solution techniques such as SAXS.

Collective conformational sampling, together with the convergence, temporal consistency, and statistical robustness of the essential-dynamics analyses, was examined in detail and is reported in the Supplementary Materials (Figures S1–S3, Tables S1–S5). Briefly, within the sampled 300 ns trajectories, the essential subspaces show internal convergence according to running and block RMSIP analyses and are substantially conserved across the four ligand-bound complexes (inter-complex RMSIP 0.72–0.80). The leading collective modes are largely non-diffusive (cosine content ≤ 0.18), with the exception of quercetin, whose PC1 retains a partially diffusive character (0.62) and whose PC1–PC2 projection suggests the sampling of two interconvertible metastable substrates. This essential-dynamics description is therefore interpreted with caution and is not used to infer quantitative state populations or exhaustive conformational sampling. Consistent with these observations, the variance distribution (Table S1) indicates that PC1 and PC2 jointly account for 53–69% of the positional variance, so the two-dimensional PC1–PC2 projection is used only for visualization, whereas the broader essential subspace is considered in the comparative analyses. The qualitative energetic trends are assessed separately from the PCA using MM-GBSA estimates and interpreted together with persistent-contact analysis.

In the Free Energy Landscape Topography, the four systems sampled energy landscapes limited by a maximum ΔG range between 10.5 kJ/mol (kaempferol) and 11.7 kJ/mol (myricetin and quercetin), with the global minimum operationally set at 0 kJ/mol in each trajectory. The position of the global minimum on the reactive coordinate differs among the complexes: Myricetin and isorhamnetin place the minimum at low values of the grid’s RMSD coordinate (0.200 and 0.213 units, respectively), while quercetin and kaempferol shift it to higher values (0.252 and 0.255 units). The RG coordinate of the global minimum, however, remains virtually unchanged around 1.985–1.995 nm in all four complexes (Table 7), consistent with the reported homogeneity of compactness. The depth and extent of the low-energy basins introduce the second discriminating axis of the topography. Isorhamnetin shows the most populated global basin in the set, with 215 grid points below the 0.5 kJ/mol minimum, followed by myricetin (185 points), quercetin (117), and kaempferol (90). The accumulation region, within ΔG ≤ 2 kJ/mol, follows a slightly different order (isorhamnetin (720) > kaempferol (703) > myricetin (619) > quercetin (592)), reflecting a flatter topography around the minimum for kaempferol and a more pronounced concentration in the other three systems. The two-dimensional projection (Figure 12) allows for a qualitative characterization of the shape of each landscape. Myricetin and isorhamnetin (Figure 12A,B) exhibit a single, well-defined energy well, with concentric low-energy contours around the global minimum and gradual barriers toward the grid periphery. Kaempferol (Figure 12D) exhibits a comparable topography, but with a more extensive low-energy zone and less compact contours, consistent with the larger population of points within ΔG ≤ 2 kJ/mol despite the lower concentration at ΔG ≤ 0.5 kJ/mol. Quercetin (Figure 12C) offers the most distinctive topographic feature, where the surface shows two partially separated low-energy regions along the RMSD coordinate, with the global minimum in one of them and a comparable depth local minimum shifted to lower coordinate values. This bimodal pattern of the energy landscape corresponds to the bimodal distribution identified in the PC1–PC2 projection (Figure S2c) and is compatible with the sampling of two interconvertible metastable substates during the 300 ns in this complex.

The integrated RMSD, PCA, and FEL analyses consistently indicate that myricetin and isorhamnetin predominantly sample a single low-energy conformational basin throughout the simulation. Kaempferol exhibits a similarly unimodal landscape, although with a broader low-energy region, whereas quercetin samples two populated low-energy regions in both the FEL and PCA projections, suggesting greater conformational heterogeneity during the simulated interval.

The values shown in the diagram (Figure 13) correspond to specific atomic interactions and, by design, are delimited by the total residue occupancies consolidated in Table 9. No direct ionic, metal coordination, or persistent π-cationic interactions were detected in any of the systems. The few isolated events (Arg57 and Arg136 as potential π-cationic acceptors in quercetin with an occupancy < 0.5%) do not exceed the relevant persistence threshold. The cooperative hinge interactions involving Glu131 and Cys133, consistent with canonical type-I kinase inhibitor interactions, was largely maintained over the 300 ns in the four complexes, but with a differential hierarchy between the two acceptors/donors. Myricetin, isorhamnetin, and quercetin favor Glu131 as a quasi-permanent contact of the 7-OH group of ring A (overall occupations of 99.80%, 99.97%, and 99.20%, respectively, reproduced in the diagram with values of 99% in all three cases; Figure 13B,D,H), while Cys133 functions as a persistent secondary acceptor on the same 7-OH with specific atomic occupations of 54% in myricetin, 46% in isorhamnetin, and 73% in quercetin (overall 58.45%, 46.12%, and 75.84%). Kaempferol completely reverses this hierarchy: In the diagram in Figure 13F, Cys133 emerges as the dominant anchor at the C4 carbonyl of ring C with a persistence of 90%, complemented by an additional π-lone-pair contact of 92%, while Glu131 disappears as a relevant direct contact (total occupation of 13.10%). This inversion appears to constitute the most marked discriminatory feature of the interaction within the series and aligns with the local rearrangement phase, identified during the first 50–100 ns of the path.

Beyond the hinge, the hydroxylation pattern of ring B of each flavonoid modulates recognition by Glu140, Lys61, and Asp194 in a manner that could be directly interpreted from ligand topology (Figure 13B,D,F,H). Kaempferol, with a single 4′-OH group on ring B, forms a single hydrogen bond with Glu140 at 71% (71.54% overall). Quercetin, with the catechol 3′,4′-dihydroxy pattern, divides this contact into two specific bonds with the same Glu140 at 68% and 81% atomic persistence, which explains the overall occupancy of 87.40%, the highest value in the series. Myricetin, with the pyrogallol 3′,4′,5′-trihydroxy pattern, distributes recognition between a double coordination with Glu140 at 71% (at the 3′-OH; total 73.51%) and a hydrogen bond with Lys61 at 59% at the 5′ hydroxyl of ring B, a unique feature of myricetin in the entire series (total occupancy of 64.81%) and a consistent with the hydroxylation pattern of the pyrogallol system. Isorhamnetin loses these perimetric contacts through methylation of the 3′-hydroxyl of ring B, eliminating the hydrogen donor at that position; its profile is reduced to the bidentate hinge anchor complemented by a hydrogen bond with Lys82 at 34% (total 34.39%). The mono-/di-/tri-hydroxy pattern of ring B appears to scale, in an approximately monotonic manner, with the number and persistence of the perimetric hydrogen bonds formed by each flavonoid. Hydrophobic stabilization of the adenine cavity is structured around Phe183 in all four complexes, with total occupancies ranging from 33.62% (isorhamnetin) to 60.41% (kaempferol) (Table 8). In all cases, the two-dimensional diagram places Phe183 in orthogonal projection onto the C ring of the flavonoid (Figure 13B,D,F,H), the canonical geometry of aromatic stacking. This π–π stacking is effective in kaempferol, myricetin, and quercetin (80%, 74%, and 71% respectively; overall 84.84%, 77.24%, and 77.01%), but is not observed in isorhamnetin, where methylation of the 3′-OH of ring B reduces the planarity of the system and degrades the contact, resulting in a generic hydrophobic interaction maintained at 75% in the diagram without the geometric stacking component. Leu59 and Ala80 complete the hydrophobic core in all four systems, with quercetin achieving maximum series occupancy at Ala80 (44.89%) and kaempferol incorporating a pronounced hydrophobic contact with Leu130 (47.35%), which does not appear in the other three complexes.

The water-bridged network suggests a second recognition layer structured around Asp194 of the DFG motif. Isorhamnetin and kaempferol maintain direct hydrogen bonds with Asp194 of 82% and 92%, respectively; myricetin partially transfers this contact to an aqueous network in which two different water molecules bridge the 3′-OH of ring B to Asp194 at 33% + 33% and the 4′-OH with Asp194 at 42% + 42%, maintaining an overall occupancy of Asp194 as a water bridge acceptor of 80.97%. Quercetin takes this pattern to the extreme: The direct bridge with Asp194 is not detected as persistent (<5%), and recognition is channeled entirely through a network of two water molecules in series, with atomic occupancies of 59% + 59% and 51% + 51%. These compounds are consolidated in an overall occupancy of Asp194 as a water bridge acceptor of 82.54%, the highest in the series. The residues Lys61, Gly180, Ser137, Leu59, and Glu140 complete the panel of peripheral residues in the aqueous network, with Lys61 being the most conserved water-mediated contact among the four flavonoids (overall occupancies of 18.86% to 30.59%). Quercetin defines a unique recognition profile within the series that deserves special attention. It is the only flavonoid in which Asp194 operates exclusively through aqueous mediation, with two structurally retained water molecules connecting the 3′-OH of ring B to the carboxylate of the residue. It is also the only complex in which Glu140 simultaneously anchors to both hydroxyl groups of the catechol (68% and 81% atomic persistence), configuring an extended hydrogen recognition, which combines the canonical hinge acceptors (Glu131 + Cys133) with bidentate peripheral anchoring on Glu140. The residue Lys82 (a residue with electrostatic function in the γ-phosphate of native ATP) also exhibits the highest occupancy in the series as a water bridge acceptor/donor (26.12%) in quercetin, compared to values ≤ 9.83% in the other three complexes. This hydrated and distributed profile is consistent with the bimodality of the described conformational ensemble and compatible with sampling two substates, in which the ligand accommodates anchoring through partially interchangeable geometries between direct and water-mediated contacts. The integration of the five contact types defines a common distinctive feature of the interaction: bidentate hinge anchoring via Glu131/Cys133, hydrophobic stabilization at Phe183, and a water-mediated peripheral network.

According to the thermodynamic affinity analysis using MM-GBSA, the four complexes showed negative and convergent ΔG_Bind_ values in the range of −49 to −55 kcal·mol^−1^, which is consistent with a thermodynamically favorable interaction along the entire pathway. The average affinity order placed the 2RKU/myricetin complex in first position (ΔG_Bind_ = −54.99 ± 5.69 kcal·mol^−1^), followed by 2RKU/kaempferol (−52.99 ± 4.45 kcal·mol^−1^), 2RKU/quercetin (−51.61 ± 4.24 kcal·mol^−1^), and 2RKU/isorhamnetin (−49.11 ± 3.61 kcal·mol^−1^). The differences between consecutive ligands in the ranking are below the individual standard deviation of each complex, so the ordering could be interpreted as a qualitative trend rather than a thermodynamic separation, which differences are within the expected uncertainty of the method.

The partitional analysis indicated differentiated unbound contributions among the four flavonoids. The van der Waals term dominated the energy landscape in all cases, with magnitudes between −34.8 and −39.6 kcal·mol^−1^, reflecting the packing of the flavone core against the hydrophobic pocket of the ATP site. The complex with kaempferol exhibited the most favorable ΔGvdW (−39.63 ± 2.36 kcal·mol^−1^), along with the most negative lipophilic term (ΔGLipo = −9.57 ± 0.95 kcal·mol^−1^), which is consistent with the high-occupancy hydrophobic contact reported with Cys133 and Phe183.

The electrostatic term (ΔG_Coulomb_) showed the greatest variance among the complexes, ranging between −20.0 to −30.4 kcal·mol^−1^. Myricetin exhibited the most negative coulombic contribution in the series (−30.40 ± 5.80 kcal·mol^−1^), which is consistent with the ability of the 3′,4′,5′-trihydroxylated pyrogallol motif of ring B to form multiple simultaneous hydrogen bonds, with Glu140 and Lys61, and with the bridge to Glu131 from the 7-OH of ring A. Quercetin, with its 3′,4′-dihydroxylated catechol, ranked second in this metric (−25.44 ± 4.34 kcal·mol^−1^), while isorhamnetin, whose 3′-methoxylation suppresses one of the two catechol donors, registered the least negative coulombic term in the series (−20.01 ± 3.20 kcal·mol^−1^). This gradation mirrors the hydroxylation pattern of ring B and is consistent with a link between the distinctive feature of the observed polar interaction and the electrostatic contribution quantified by MM-GBSA.

The explicit hydrogen bonding term (ΔG_HBond_) followed a trend consistent with the coulombic pattern, with values between −2.28 kcal·mol^−1^ (isorhamnetin) and −3.19 kcal·mol^−1^ (myricetin), which is consistent with the interpretation that the number and persistence of available hydroxyl donors on ring B are associated with increased polar contribution to the bond.

The polar desolvation penalty (ΔG_Solv-GB_) ranged from +19.5 to +23.8 kcal·mol^−1^ and correlated directly with the magnitude of the coulombic term associated with each complex: Myricetin, which maximizes polar contacts with the receptor, also experiences the most costly desolvation (+23.82 ± 2.46 kcal·mol^−1^), while isorhamnetin experiences the lowest penalty (+19.48 ± 1.97 kcal·mol^−1^). The partial compensation between ΔG_Coulomb_ and ΔG_Solv-GB_ represents the expected thermodynamic equilibrium for polar interactions in an aqueous environment and explains why the net affinity gain of the larger ring-B hydroxylation pattern is modest, compared to the absolute magnitude of the electrostatic term.

The dispersion of ΔGBind between frames, measured as a standard deviation, was minimal in isorhamnetin (±3.61 kcal·mol^−1^) and maximal in myricetin (±5.69 kcal·mol^−1^). These differences indicate ligand-dependent fluctuations in the MM/GBSA estimates; however, they should not be interpreted as a direct proxy for conformational heterogeneity, because the energetic dispersion and the collective motions captured by PCA describe distinct aspects of the sampled trajectories.

The sum of the non-polar, lipophilic, and packing contributions was essentially comparable among the four complexes (with values ranging from −45 to −50 kcal·mol−1 for ΔGvdW + ΔGLipo + ΔGPacking combined), suggesting that the flavone core establishes a conserved contact pattern with the 2RKU hydrophobic pocket among the four ligands, while the differential modulation of affinity is concentrated in the polar terms (Coulomb, HBond, Solv-GB). This partitioning is consistent with the mechanistic interpretation that the hydroxylation pattern of ring B is the main structural-chemical determinant of the differential affinity with similar dispersive contributions across the flavone scaffold.

## 4. Materials and Methods

### 4.1. QSAR Modeling

#### 4.1.1. PLK-1 Inhibitor Dataset

The dataset used in this study consists of small molecules from the ChEMBL chemical database [60], obtained from their corresponding SMILE code (available in the Supplementary Information). Experimental data on the maximum inhibitory concentration at 50% (IC_50_ in nM) expressed as pIC_50_ (−log IC_50_ in M), for the PLK-1 enzyme, were used as the response variable.

#### 4.1.2. Calculation of Molecular Descriptors

All the molecular descriptor families implemented in DRAGON software version 7.0 [61] are based on a three-dimensional representation of the molecules, due to the stereospecificity of the enzymes, through optimization using the semi-empirical AM1 method [62] implemented in MOPAC [63]. It was decided to use only one conformation generated by the method, without verifying the frequencies, since the nature of the stationary point in the inhibitor-enzyme interaction is unknown.

#### 4.1.3. Data Pre-Processing

For data pre-processing, variables with missing, constant, or nearly constant values, as well as those with a 95% correlation coefficient, were removed. Additionally, a hierarchical cluster analysis was implemented in STATISTICA v6 [64] to identify duplicates and potential outliers.

#### 4.1.4. Discretization of the Response Variable

To discretize the response variable, the procedure described previously [65,66] was followed, using a piecewise regression method implemented in STATISTICA. In this method, two independent linear regression equations were estimated (Equation (4)), one for *y*-values less than or equal to the breakpoint (*b*_0_) and another for y-values greater than the breakpoint. The critical pIC_50_ value (breakpoint) was established and estimated using the program where the data behavior differed. Based on these results, two classification groups or classes were formed (active class or strong inhibitors, and inactive class or weak inhibitors).

(4) $$y=\left(b_{01}+{\;b}_{11}x_{1}+\ldots +b_{m1}x_{m}\right)\left(y\;\leq \;b_{n}\right)+\left(b_{02\;}+b_{12}x_{1}+\ldots +{\;b}_{m2}x_{m}\right)\left(y\;>\;b_{n}\right)$$

#### 4.1.5. Selection of Training and Prediction Series

To assess the distribution and structural diversity within the observation groups, a *k*-means cluster analysis was performed for each class using the STATISTICA program [64]. From this study, 25% of the compounds in each cluster were selected for the prediction set (PS), and the remaining subset constituted the training set (TS).

#### 4.1.6. Variable Selection

In this work, a genetic algorithm, an artificial intelligence technique, was used to select an optimal population of variables for regression, with 10,000 iterations. In addition, exhaustive and sequential exponential search techniques, such as optimal subset and forward stepwise, were used to select the statistically most significant variables in the qualitative modeling.

#### 4.1.7. Obtaining the Classification Models

The linear discriminant analysis model was developed using the STATISTICA program to discriminate between two classes (strong PLK-1 inhibitors and weak PLK-1 inhibitors), seeking the linear combination of independent variables that best allows differentiating (discriminating) the groups, according to Equation (5):

D = a + b1 × x1 + b2 × x2 + … + bm × xm(5)

where a is a constant and b1–bm are the coefficients of the function.

The multilayer perceptron (MLP) network model was developed using the STATISTICA program. A general (9:n:2) architecture was used, including the preselected variables as input neurons and two output neurons corresponding to the classes. The BFGS, Scaled Conjugate, and Gradient Descent training algorithms were tested to obtain the models. Furthermore, a range of activation function options were explored for the hidden neurons (identity, hyperbolic tangent (tanh), logistic sigmoid, exponential, and sine) and for the output neurons (identity, tanh, logistic sigmoid, exponential, sine, and softmax), using the sum of squares error (SSE) function in Equation (6) or the cross-entropy (CE) error function in Equation (7):

(6) $$E_{\mathrm{SOS}\;}=\;\sum_{i=1}^{N}{\left(y_{i}-t_{i}\right)}^{2}$$

(7) $$E_{\mathrm{CE}}=-\sum_{i=1}^{N}t_{i}\ln\left(\frac{y_{i}}{t_{i}}\right)$$

where N is the number of training cases and y_i_ is the prediction (network outputs) of the target value t_i_ for the i-th data case.

#### 4.1.8. Validation

To evaluate the quality and robustness of the obtained discriminant models, several statistical parameters were analyzed, such as Wilks’ λ, the F value, and the square of the Mahalanobis distance (D^2^). Another factor considered to evaluate the capacity of the obtained classification functions was the percentage of correct classification (Accuracy, Q) for all models in the TS.

Similarly, the statistical parameters recommended in the medical-statistical literature were calculated. In this regard, the quality of the models obtained was also expressed through the Matthews correlation coefficient (C), sensitivity (Sens), specificity (Spec), and the false positive rate (FPR) according to Equations (8)–(12) [67]:

(8) $$Q\;=\;100\;\times \;\frac{\left(\mathrm{TP}\;+\;\mathrm{TN}\right)}{\left(\mathrm{TP}\;+\;\mathrm{FP}\;+\;\mathrm{TN}\;+\;\mathrm{FN}\right)}$$

(9) $$C=100\;\times \;\frac{\left(\mathrm{TP}\;\times \;\mathrm{TN}-\mathrm{FP}\;\times \;\mathrm{FN}\right)}{\sqrt{\left(\mathrm{TN}+\mathrm{FN}\right)\times \left(\mathrm{TN}+\mathrm{FP}\right)\times \left(\mathrm{TP}+\mathrm{FP}\right)\;\times \;\left(\mathrm{TP}+\mathrm{FN}\right)}}$$

(10) $$\mathrm{Sens}=100\;\times \;\frac{\mathrm{TP}}{\left(\mathrm{TP}+\mathrm{FN}\right)}$$

(11) $$\mathrm{Spec}=100\;\times \;\frac{\mathrm{TP}}{\left(\mathrm{TP}+\mathrm{FP}\right)}$$

(12) $$\mathrm{FPR}=100\;\times \;\frac{\mathrm{FP}}{\left(\mathrm{FP}+\mathrm{TN}\right)}$$

where TP and TN represent true positives and negatives, respectively, and FP and FN are the false positives and negatives, respectively.

Furthermore, the AMBIT Discovery software v0.04 [68] was used to define the model’s AD, a free and easy-to-use application. In this process, the similarity of each compound to the chemical space defined by the descriptors was calculated, taking into account Euclidean and Cityblock distances [69].

#### 4.1.9. Virtual Screening

The resulting models were used for the virtual screening of a set of 27 flavonoids present in food, described by the Beltsville Human Nutrition Research Center in 2015 [49].

The selection criteria considered were: (i) The selected compounds were predicted to be active (inhibitors) by both classification models, (ii) They lie within the theoretical region of the chemical space defined by the model descriptors and the modeled response (applicability domain), to ensure the reliability of the predictions, and (iii) They meet all the considerations of Lipinski’s rule of five [70] for oral bioavailability.

### 4.2. Docking

#### 4.2.1. Software and Computational Resources

All calculations were performed using the Schrödinger Suite 2020-3 software package (Maestro, Protein Preparation Wizard, Epik, LigPrep, Glide, and Prime; Schrödinger, LLC, New York, NY, USA). Unless otherwise stated, default parameters were used. Energy minimization and binding free energy calculations were performed using the OPLS3e force field [71], and solvation effects were modeled using the implicit solvent VSGB 2.0 [72].

#### 4.2.2. Receptor Selection and Preparation

The selected receptor structure was the human Polo-like Kinase 1 (PLK1-KD) kinase domain (PDB 2RKU) [46], co-crystallized with the type-I inhibitor BI-2536. This entry was chosen according to pre-specified criteria: (i) high resolution (1.95 Å; threshold ≤ 2.0 Å), (ii) favorable wwPDB validation metrics (R_work/R_free = 0.190/0.241; clashscore = 4; >99% model completeness), and (iii) the presence of a relevant co-crystallized ligand, which defines the canonical hinge-binding mode typical of competitive ATP inhibitors [73]. The receptor was prepared using the Protein Preparation Wizard [52], which included assigning bond orders, adding hydrogen atoms, optimizing hydrogen bond networks, adjusting protonation states to physiological pH, and creating disulfide bonds when necessary. Subsequently, all crystallographic water molecules located more than 5 Å from the co-ligand were removed. Finally, constrained minimization was performed under the OPLS3e force field until convergence with a heavy atom RMSD of 0.30 Å to relieve local steric strain without altering the native geometry of the active site.

#### 4.2.3. Ligand Preparation

The flavonoids myricetin, isorhamnetin, quercetin, and kaempferol, as well as the reference ligand BI-2536, were processed using LigPrep (Schrödinger Suite 2020-3). Two-dimensional structures were converted to low-energy three-dimensional conformers, and the most probable ionization and tautomeric states at pH 7.0 ± 2.0 were predicted using Epik [74]. Relevant stereoisomers were conserved to ensure biologically plausible bond.

#### 4.2.4. Grid Generation and Docking Protocol Validation

A receptor grid was generated using Glide [45], centered on the centroid of the BI-2536 molecule co-crystallized in 2RKU, encompassing the ATP-binding site and the hinge region. The docking workflow was validated by redocking BI-2536 into the prepared receptor. The protocol was considered reliable when the redocked conformation reproduced the crystallographic orientation, with a root mean square deviation (RMSD) < 2.0 Å for the heavy atoms, maintaining the hydrogen bonds characteristic of the hinge region of ATP-binding site inhibitors [75].

#### 4.2.5. Hierarchical Docking: SP Screening and XP Refinement

Molecular docking was performed using a hierarchical flexible-ligand/rigid-receptor workflow. First, the co-crystallized BI-2536 was redocked using Standard Precision (SP) mode to ensure accurate reproduction of the experimental binding mode. Subsequently, the same SP procedure was applied to BI-2536 and all flavonoids to explore thoroughly the conformational and positional spaces of the ATP-pocket. All conformations generated by SP were refined using the Extra Precision (XP) algorithm, which applies stricter steric penalties and incorporates a hydrophobic enclosure model [50]. This sequential SP→XP approach is consistent with established practices for achieving comprehensive sampling and accurate classification in kinase docking studies.

#### 4.2.6. MM/GBSA Binding-Free Energy Calculations

All conformations refined using XP were subsequently analyzed using the MM/GBSA method implemented in Prime (Schrödinger Suite 2020-3) [76]. Calculations were performed using the OPLS3e and VSGB solvation models. Complexes were minimized using the REAL_MIN option, and an energy analysis was requested to decompose ΔG_bind_ into ΔG_bind_(NS) (without strain), as well as into the strain components of the ligand and receptor. This procedure provides thermodynamically consistent estimates of binding affinity and allows for the evaluation of strain penalties associated with each ligand conformation.

#### 4.2.7. Selection of the Final Pose

The final representative complexes were selected using a consensus approach that integrated three independent metrics: (i) SP coupling GlideScore, (ii) XP refinement GlideScore, and (iii) MM/GBSA binding-free energy (ΔG_bind_ and ΔG_bind_(NS)). Only those conformations that consistently obtained favorable values on all three criteria and reproduced the hinge hydrogen bonding interactions observed in the BI-2536–PLK1 crystal complex were retained as plausible binding modes. This strategy minimizes dependence on any single scoring function and improves the reproducibility of the binding mode assignment.

### 4.3. Molecular Dynamics

#### 4.3.1. System Preparation and Parameterization

The molecular docking poses of the protein–ligand complexes 2RKU/isorhamnetin, 2RKU/kaempferol, 2RKU/myricetin, and 2RKU/quercetin were used as initial structures for the molecular dynamics simulations. The initial topology and coordinates of the systems were constructed using the OPLS3e force field [71], via the System Builder module of the Schrödinger suite (version 2020-3). Each complex was solvated in an orthorhombic box using the explicit water model TIP3P [77], establishing a minimum buffer margin of 10.0 Å in all spatial directions. To neutralize the system and mimic physiological conditions, counterions were added, and a saline concentration of 0.15 M NaCl was set.

#### 4.3.2. Energy Minimization and Thermal Relaxation Protocol

The solvated systems underwent initial conditioning consisting of a rigorous 2000-step energy minimization, with a gradient convergence threshold of 1.0 kcal/(mol·Å). Subsequently, a multi-stage relaxation protocol was executed, beginning with constant particle number, volume, and temperature (NVT) assembly Brownian dynamics at 10 K for 100 ps, applying harmonic constraints (force constant of 50.0 kcal/(mol·Å^2^)) to the heavy solute atoms. Thermal conditioning was performed using a discrete heating ramp divided into four target windows: 10 K, 100 K, 200 K, and 300 K. This ramp was managed by a Nosé-Hoover chain thermostat [78] with a relaxation time of 1.0 ps. After reaching 300 K, the protocol transitioned to a constant particle number, pressure, and temperature (NPT) assembly equilibration at 1.01325 bar, using the Martyna-Tobias-Klein barostat to stabilize the density before releasing the structural constraints.

#### 4.3.3. Molecular Production Dynamics

Production simulations were run with GPU acceleration using the Desmond engine. Each system was simulated for a continuous period of 300 ns (300,000 ps) in the NPT assembly. The temperature was held constant at 300 K and the pressure at 1.01325 bar (1 atm), coupled using Langevin dynamics. The equations of motion were solved using the RESPA multi-step integrator algorithm [79], with integration steps of 2.0 fs for short-range bound and unbound interactions, and 6.0 fs for long-range electrostatic forces. Short-range van der Waals and coulombic interactions were truncated to a cutoff radius of 9.0 Å, while long-range interactions were calculated using the particle mesh Ewald (PME) method [80]. The coordinates were stored every 100.0 ps, generating a set of 3000 frames per trajectory.

#### 4.3.4. Trajectory Analysis and Conformational Dynamics

Protein–ligand interaction and basic stability analyses (RMSD and RMSF) were performed using Schrödinger’s event_analysis.py script. To analyze further the thermodynamic conformational sampling of each system, a post-dynamic analysis was implemented using the G_Measures v.0.9d plugin in PyMOL v2.0 [81]. Structural compactness was assessed using the radius of gyration (RG). A principal component analysis (PCA) was also performed, constructing a 10-component model to project significant collective motions. Sampling convergence of the collective modes was assessed from the cosine content of the principal components [82]; values near zero indicate converged sampling, whereas values approaching unity are characteristic of random diffusion. Finally, a free energy landscape (FEL) was constructed by deriving the Gibbs free energy as a function of the structural RMSD and the RG, to identify the metastable conformational states traversed during the 300 ns.

#### 4.3.5. Binding-Free Energy Calculations (MM/GBSA)

Thermodynamic affinity was quantified, using the Born Molecular Mechanics/Generalized Solvent Accessible Surface Area (MM/GBSA) method [83,84]. The thermal_mmgbsa.py script, coupled to the Prime module [85], was used to perform the calculations, processing a total of 3000 conformations (frames) per complex, extracted every 100 ps from the production pathways. Energy evaluation was based on the VSGB 2.0 implicit solvation model [72] and the OPLS3e force field. Flexibility was treated using the real minimization mode (REAL_MIN), maintaining a flexibility radius of 0.0 Å with respect to the ligand.

## 5. Conclusions

The LDA and MLP classification models captured structure–activity relationships associated with PLK-1 inhibition and achieved predictive accuracies above 85%, supporting their use for virtual screening within the defined applicability domain. Myricetin, isorhamnetin, quercetin, and kaempferol were prioritized as leading flavonoid candidates and showed favorable predicted interactions and binding energetics at the PLK-1 active site. Their recognition involves a conserved interaction network in the ATP-binding pocket, including hinge-region contacts with Glu131 and/or Cys133 and hydrophobic packing against the adenine cavity, while ligand-specific differences are associated with the B-ring substitution and hydroxylation pattern. Among the evaluated compounds, myricetin showed the most favorable mean MM/GBSA estimate, associated with stronger Coulombic contributions and a distinctive hydrogen-bond topology; however, the overlap in energetic distributions warrants cautious interpretation of inter-ligand differences rather than a definitive affinity ranking. The 300 ns molecular dynamics analyses further support preservation of the kinase-domain fold and persistence of key binding interactions across the sampled trajectories. Because one production trajectory was analyzed per complex, these dynamic results are interpreted as evidence of sampled structural stability and binding-mode persistence rather than exhaustive conformational sampling or quantitative state populations. The integrated ligand- and structure-based workflow supports the prioritization of these flavonoids for experimental evaluation. Future work should include direct *in vitro* PLK-1 inhibition assays to determine IC_50_ values, followed by cell-based studies of proliferation, migration/invasion, and relevant metastasis-associated markers, and ultimately *in vivo* validation of the most promising candidates.

### Key Findings and Perspective

Overall, this study presents an integrated computational strategy combining ligand-based machine learning, molecular docking, molecular dynamics simulations, and MM/GBSA binding free-energy calculations into a unified computational workflow for the prioritization of potential PLK-1 inhibitors. Among the evaluated flavonoids, myricetin, isorhamnetin, quercetin, and kaempferol emerged as the highest-priority candidates, providing a rational basis for their future experimental evaluation.

Given that tumor metastasis is a major determinant of poor patient prognosis, the identification of novel therapeutic strategies capable of preventing or limiting metastatic progression remains a critical challenge. In this context, the computational workflow presented here may facilitate the early prioritization of anti-metastatic candidates during the initial stages of the drug discovery and development process, thereby potentially reducing early-stage drug-discovery costs and minimizing the unnecessary use of experimental animals while guiding subsequent experimental validation.

## Figures and Tables

**Table 1:** Performance of the MLP model.

	N	C	Q (%)	Sens (%)	Spec (%)	FPR	AUC
TS	359	0.751	88.301	92.308	89.083	18.116	0.923
PS	120	0.812	90.833	94.286	90.411	14.000	0.933

**Table 2:** Virtual screening results.

Flavonoids	ΔP ^a^	LDA ^b^	MLP ^c^	LAI ^d^	AD
01 Isorhamnetin	0.8638	+	+	0	In
02 Kaempferol	0.8502	+	+	0	In
03 Myricetin	0.8824	+	+	0	In
04 Quercetin	0.8572	+	+	0	In
05 Apigenin	0.4238	+	+	0	In
06 Luteolin	0.4910	+	+	0	In
07 Eriodictyol	0.4680	+	+	0	In
08 Hesperetin	0.4540	+	+	0	In
09 Naringenin	0.4331	+	+	0	In
10 (+)-Catechin	−0.5881	−	−	0	In
11 (+)-Catechin-3-gallate	0.1952	+	−	0	In
12 (-)-Epicatechin	−0.5461	−	−	0	In
13 (-)-Epicatechin-3-gallate	0.4771	+	−	0	In
14 (-)-Epigallocatechin	−0.7526	−	−	0	In
15 (-)-Epigallocatechin-3-gallate	0.2962	+	−	0	In
16 (+)-Gallocatechin	−0.6423	−	−	0	In
17 (+)-Gallocatechin-3-gallate	0.4176	+	−	0	In
18 Theaflavin	−0.8755	−	−	1	Out
19 Theaflavin-3-gallate	−0.8790	−	−	1	Out
20 Theaflavin-3′-gallate	−0.9774	−	−	1	Out
21 Theaflavin-3,3′-digallate	−0.9714	−	−	1	Out
22 Cyanidin	0.3641	+	+	0	In
23 Delphinidin	0.4198	+	+	0	In
24 Malvidin	0.3274	+	+	0	In
25 Pelargonidin	0.3310	+	+	0	In
26 Peonidin	0.3799	+	+	0	In
27 Petunidin	0.4143	+	+	0	In

^a^ ΔP = (probability of belonging to the active class) − (probability of belonging to the inactive class). ^b^ Class predicted by the LDA model. ^c^ Class predicted by the MLP model. ^d^ The Lipinski Alert Index (LAI) is a molecular descriptor, which takes the value 0 if it meets all of Lipinski’s criteria for oral absorption.

**Table 3:** Docking results.

Ligand	SP Docking Score	XP Glide Score	MM/GBSA ΔG_bind_ (kcal·mol^−1^)
BI-2536 (co-cristal, 2RKU)	−13.289	−13.289	−98.81
Myricetin	−10.403	−10.444	−51.68
Kaempferol	−10.193	−10.229	−41.11
Quercetin	−9.741	−9.777	−45.34
Isorhamnetin	−8.969	−9.005	−45.93

**Table 4:** RMSD of the Cα complexes in the equilibrium window (200–300 ns).

Complex	Mean RMSD (Å)	σ (Å)	Maximum in Plateau (Å)
2RKU-Myricetin	1.996	0.125	2.572
2RKU-Isorhamnetin	2.052	0.197	2.651
2RKU-Quercetin	2.352	0.179	2.781
2RKU-Kaempferol	2.587	0.178	3.155

**Table 5:** Specific flexibility of residues in 2RKU–flavonoid complexes.

Parameter	Myricetin	Isorhamnetin	Kaempferol	Quercetin
Mean RMSF (Å)	0.911	0.910	1.033	1.087
Median RMSF (Å)	0.762	0.726	0.827	0.875
Residues with RMSF < 1.0Å (%)	75.9	75.9	65.3	57.1
Glu131—hinge (Å)	0.579	0.610	0.639	0.756
Cys133—hinge (Å)	0.661	0.656	0.667	0.650
Phe183—adenine cavity¬ (Å)	0.502	0.501	0.563	0.558
Lys82—catalytic (Å)	0.666	0.588	0.603	0.836
Asp194—DFG motif (Å)	0.663	0.511	0.502	0.652

**Table 6:** Radius of gyration of the PLK-1 kinase domain in 2RKU-flavonoid complexes.

2RKU-Flavonoid Complex	Medium RG 0–300 ns (Å)	σ (Å)	RG Mean 200–300 ns (Å)	RG Range (Å)
Kaempferol	19.862	0.098	19.841	19.553–20.233
Myricetin	19.927	0.112	19.875	19.564–20.345
Isorhamnetin	19.993	0.101	19.981	19.704–20.491
Quercetin	20.018	0.102	20.019	19.712–20.428

**Table 7:** Topographic parameters of the two-dimensional free energy landscape for 2RKU-flavonoid complexes.

Parameter	Myricetin	Isorhamnetin	Kaempferol	Quercetin
Maximum grid ΔG (kJ/mol)	11.7	11.6	10.5	11.7
RMSD coordinate of the global minimum (a.u.)	0.200	0.213	0.255	0.252
RG coordinate of the global minimum (nm)	1.985	1.995	1.987	1.995
Points with ΔG ≤ 0.5 kJ/mol	185	215	90	117
Points with ΔG ≤ 2 kJ/mol	619	720	703	592

**Table 8:** Occupancy matrix (%) of persistent protein–ligand contacts for 2RKU-flavonoid complexes.

Type/Residue	Myricetin	Isorhamnetin	Kaempferol	Quercetin
**Hydrogen bonds**				
Glu131	99.80	99.97	13.10	99.20
Cys133	58.45	46.12	97.33	75.84
Asp194	17.69	82.54	92.44	—
Glu140	73.51	—	71.54	87.40
Lys61	64.81	6.53	—	—
Ser137	30.06	—	—	21.39
Lys82	5.06	34.39	—	—
Cys67	—	—	7.43	14.13
Leu59	5.70	8.80	—	—
Arg136	7.46	—	—	—
**Hydrophobic contacts**				
Phe183	36.79	33.62	60.41	40.45
Ala80	35.25	25.99	—	44.89
Leu59	21.13	7.76	20.16	18.83
Leu130	—	10.10	47.35	—
Cys67	—	8.80	—	—
Val114	—	5.40	—	—
π–π stacking				
Phe183	77.24	—	84.84	77.01
**Water-mediated bridges**				
Asp194	80.97	63.95	20.86	82.54
Lys61	20.36	30.49	30.59	18.86
Glu140	12.20	33.29	8.50	7.60
Gly180	18.16	—	—	16.96
Leu59	13.43	11.20	11.80	17.19
Ser137	7.53	14.13	20.39	11.16
Lys82	9.83	7.66	—	26.12
Arg136	7.70	21.66	—	—
Glu101	—	23.39	—	—

**Table 9:** Energy decomposition of 2RKU-flavonoid complexes using MM-GBSA.

Complex	ΔG_Bind_	ΔG_Coulomb_	ΔG_HBond_	ΔG_Lipo_	ΔG_Solv-GB_	ΔG_vdW_
2RKU/Isorhamnetin	−49.11 ± 3.61	−20.01 ± 3.20	−2.28 ± 0.54	−7.47 ± 0.92	+19.48 ± 1.97	−37.58 ± 2.52
2RKU/Kaempferol	−52.99 ± 4.45	−21.18 ± 4.01	−2.95 ± 0.47	−9.57 ± 0.95	+21.66 ± 1.89	−39.63 ± 2.36
2RKU/Myricetin	−54.99 ± 5.69	−30.40 ± 5.80	−3.19 ± 0.91	−8.43 ± 1.03	+23.82 ± 2.46	−34.84 ± 2.81
2RKU/Quercetin	−51.61 ± 4.24	−25.44 ± 4.34	−2.64 ± 0.64	−7.90 ± 1.09	+21.56 ± 2.18	−35.05 ± 2.50

## Data Availability

The original contributions presented in the study are included in the article/Supplementary Materials, further inquiries can be directed to the corresponding authors.

## Supplementary Materials

The following supporting information can be downloaded at https://www.mdpi.com/article/10.3390/ijms27156821/s1. References [86,87,88,89,90,91,92,93,94,95,96,97,98,99,100] are cited in Supplementary Materials.

## Abbreviations

The following abbreviations are used in this manuscript:

AD	Applicability domain
AM1	Austin model 1
ANNs	Artificial neural networks
APC/C	Anaphase promoting complex/cyclosome
ATP	Adenosine triphosphate
AUC	Area under the curve
CD	Catalytic domain
CDK1	Cyclin-dependent kinase 1
DFG	Asp-Phe-Gly
FEL	Free energy landscape
IC_50_	Maximum inhibitory concentration at 50%
LDA	Linear discriminant analysis
MLP	Multilayer perceptron
MM/GBSA	Molecular Mechanics/Generalized Born Surface Area
NVT	Constant number, volume, temperature ensemble
NPT	Constant number, pressure, temperature ensemble
OPLS3e	Optimized potentials for liquid simulations 3e
PBD	Polo-box domain
PC	Principal component
PLK1	Polo-like kinase 1
PS	Prediction set
QSAR	Quantitative structure-activity relationship
RG	Radius of gyration
RMSD	Root mean square deviation
RMSF	Root mean square fluctuation
ROC	Receiver operating characteristic
SAR	Structure-activity relationship
SID	Simulation interactions diagram
SP	Standard precision
TS	Training set
VdW	Van der Waals
XP	Extra precision
