# Supplementary material for: Identification of Natural Flavonoids Targeting PLK-1 as Potential Anti-Metastatic Agents: A Computational Approach
Source: Int J Mol Sci. 2026 Jul 29;27(15):6821. doi: 10.3390/ijms27156821 (PMC13466354; doi:10.3390/ijms27156821)
Supplement: Supplementary file 1 [file ijms-27-06821-s001.zip › Supplementary Information.pdf]

Supporting Information

Identification of Natural Flavonoids Targeting PLK-1 as Potential Anti-Metastatic Agents: A Computational Approach

Yudith Cañizares-Carmenate[1, ¥, *], Erix W. Hernández-Rodríguez[1, ¥], Yunier Perera-Sardiña[2], Dina B. Aguado-Herrera[3], Roberto Díaz-Amador[1], Francisco Torrens[4], Juan A. Castillo-Garit[5,*]

Table of Contents

		Page
Section S1	Detailed Structure-Based Analysis of Flavonoid–PLK-1 Complexes	2
Section S2	Essential-dynamics convergence, reproducibility and robustness	3
Section S2.1	Dimensionality of the essential subspace	4
Section S2.2	Two-dimensional projection of the conformational ensemble	4
Section S2.3	Convergence of the essential subspace	5
Section S2.4	Reproducibility across independent complexes	6
Section S2.5	Statistical reliability of the principal-component time series	6
Section S2.6	Robustness of the conformational states	7
Section S2.7	Inter-ligand comparison and structure–activity link	8

Section S1. Detailed Structure-Based Analysis of Flavonoid–PLK-1 Complexes

The 3'-OH→ 3'-OMe substitution between quercetin and isorhamnetin does not penalize affinity, despite the loss of the H-bond with Cys133. The compensation occurs through three quantifiable mechanisms in the docked pose: shortening of the H-bond with Glu131 ($\Delta d = 0.155$ Å), strengthening of the π-cation with Lys82 (ΔCoul = -0.254 kcal/mol), and the incorporation of an exclusive salt bridge with Glu69 (Coul = -4.192 kcal/mol) at the leading edge. The methoxyl group at the 3'-position thus acts as a biocompatible 3'-OH isoster for PLK-1, a finding of pharmacokinetic relevance, given that methylation reduces susceptibility to phase-II glucuronidation/sulfation and improves membrane permeability, compared to polyhydroxylated flavonols.

The rank inversion between GlideScore and MM/GBSA observed in kaempferol reveals, that bidentate hinge anchoring (Glu131 + Cys133) is a strict energy requirement of the type-I bonding mode, in PLK-1, not a geometric preference. Although the individual H-bond with Cys133 reaches the highest magnitude in the series in kaempferol (E_{int} = -8,349 kcal/mol), the electrostatic repulsion with Glu131 (Coul = +2,719 kcal/mol) negatively impacts the overall balance. The empirical Glide function tends to overweight intense point contributions, whereas the MM/GBSA score, by incorporating implicit solvation and non-additive electrostatic terms, adequately captures the energy cost of the collision with Glu131. This discrepancy supports the operational convenience of hierarchical SP–XP–MM/GBSA pipelines in virtual kinase screening, systematically recommended in recent computational literature [1-3].

The VdW contribution of the π-stack in Phe183 follows a decreasing trend with the polar richness of ring B: quercetin (-5,454 kcal/mol) > myricetin (-5,214) > isorhamnetin (-5,000) > kaempferol (-3,866). The approximately 30% drop in aromatic contribution between quercetin and kaempferol indicates that the monohydroxylated ring B is displaced, outside the lateral aromatic patch. This correlation establishes a geometric interdependence between the hydroxyl decoration of ring B and the orientation of the aromatic plane in Phe183: The hydroxyls of ring B not only provide direct polar engagement with the leading edge, but also orient the ligand toward the aromatic subpocket, simultaneously optimizing polar and hydrophobic contributions. This dual contribution has been documented for this same family of scaffolds compared to Pim-1 and DYRK1A [4].

The identity of the structural mediator of the water molecule differs between the reference ligand and the flavonoid series. The BI-2536 maintains crystallographic contacts with HOH_563 and HOH_527 (H-bond ≈ -1,000 kcal/mol each), while the four flavonoids converge on

HOH_698 as the dominant bridging water molecule, with an exceptional magnitude in kaempferol (H-bond = -4,471; Eint = -4,042 kcal/mol) and moderate contributions in the remaining congeners. This flavonoid-specific hydration site suggests that polyphenols exploit a lateral polar subpocket that optimized synthetic inhibitors do not take advantage of, a finding that opens a clear pathway for structure-guided derivatization: Displacement of HOH_698, by means of a B-ring substituent capable of directly interacting with the acceptor residue of the underlying protein, should increase affinity without compromising selectivity, in line with the water molecule displacement strategy validated for other kinase systems [5].

The structural and thermodynamic descriptors, taken together, establish a direct correlation between the bidentate hinge anchor (Glu131 + Cys133) and the affinity calculated by MM/GBSA, a widely recognized requirement for type-I binding mode and consistently reported in clinically evaluated PLK-1 inhibitor series [6,7]. Hydrophobic packing at Leu59 and π-stacking at Phe183 form the aromatic-aliphatic axis, which stabilizes the adenine pocket in all analyzed complexes, while structural water molecules (HOH_698 in flavonoids; HOH_563/527 in BI-2536) act as conformational mediators of the polar networks [5,8]. The convergence between the prioritization derived from the ligand-based QSAR model and the findings of the structure-based docking analysis reinforces the selection of myricetin, quercetin, and isorhamnetin as primary hits for direct biochemical validation of PLK-1, while the discrepancy between docking and MM/GBSA observed in kaempferol justifies its deprioritization, despite its good performance in GlideScore. Recent computational campaigns on PLK-1, which integrates structure-based virtual screening with molecular dynamics and biochemical validation, have reported active inhibitors with MM/GBSA energies in ranges comparable to those described here [3,9], further supporting the translational relevance of the prioritized hits as starting points for subsequent optimization lead compounds.

Section S2. **Essential-dynamics convergence, reproducibility and robustness**

This section describes the descriptive and validation analyses of the essential dynamics that support, but are not required for, the conclusions of the main text. All quantities were computed on the four PLK-1–flavonoid production trajectories (294 Cα atoms; 3000 frames; 300 ns per complex). Reference numbers refer to the main-text reference list. Because a single 300 ns trajectory is expected to undersample the slowest collective modes, these analyses are used to characterise the direction and convergence of the collective motion and to place the four

complexes in a common comparative frame, rather than to derive quantitative state populations; the affinity ranking is governed by the MM-GBSA energetics (main text, Section 2.5.6).

S2.1. Dimensionality of the essential subspace

The distribution of positional variance among the principal components was examined to establish how many collective coordinates are required to describe the conformational ensemble. As summarised in Table S1, the first two components jointly account for 53–69% of the variance and the first three for 64–76%, so no system reaches the 80% threshold within three components. Quercetin concentrates the largest fraction on PC1 (49.9%) and shows the widest projection range (5.86 a.u.), whereas myricetin is the most evenly distributed (PC1 = 33.7%). These values suggest that a PC1–PC2 description is informative for visualisation but insufficient as a quantitative basis; accordingly, the convergence and clustering analyses below were performed in a five-component subspace rather than in PC1–PC2 alone [10]. The per-component variance scree and the cosine-content profile are shown in the authors' pre-existing Figure S1.

Table S1. *Conformational variance distribution of the ensemble (moved from the main text): percentage of positional variance captured by the leading principal components and projection range onto PC1.*

Parameter	Myricetin	Isorhamnetin	Kaempferol	Quercetin
**Variance PC1 (%)**	33.7	39.9	37.3	49.9
**Variance PC2 (%)**	19.5	15.1	17.6	18.6
**Variance PC1+PC2 (%)**	53.2	55.0	54.9	68.6
**Variance PC1+PC2+PC3 (%)**	64.3	66.4	66.2	75.6
**Projection range onto PC1 (a.u.)**	4.94	4.57	5.44	5.86

S2.2. Two-dimensional projection of the conformational ensemble

The projection of each trajectory onto its first two principal components provides a qualitative picture of the sampled conformational space (Figure S1). Myricetin and isorhamnetin (panels A and B) explore a single, relatively compact cloud, consistent with a homogeneous bound state. Kaempferol (panel D) samples a comparable but slightly broader region. Quercetin (panel C) displays the widest and most clearly bipartite distribution along PC1, which is consistent with its higher PC1 variance (Table S1) and with the partially diffusive character of its leading mode (Table S2). This behaviour appears to reflect the sampling of two interconvertible substates and is mirrored by the bimodal free-energy landscape of quercetin discussed in the main text (Section 2.5.4).

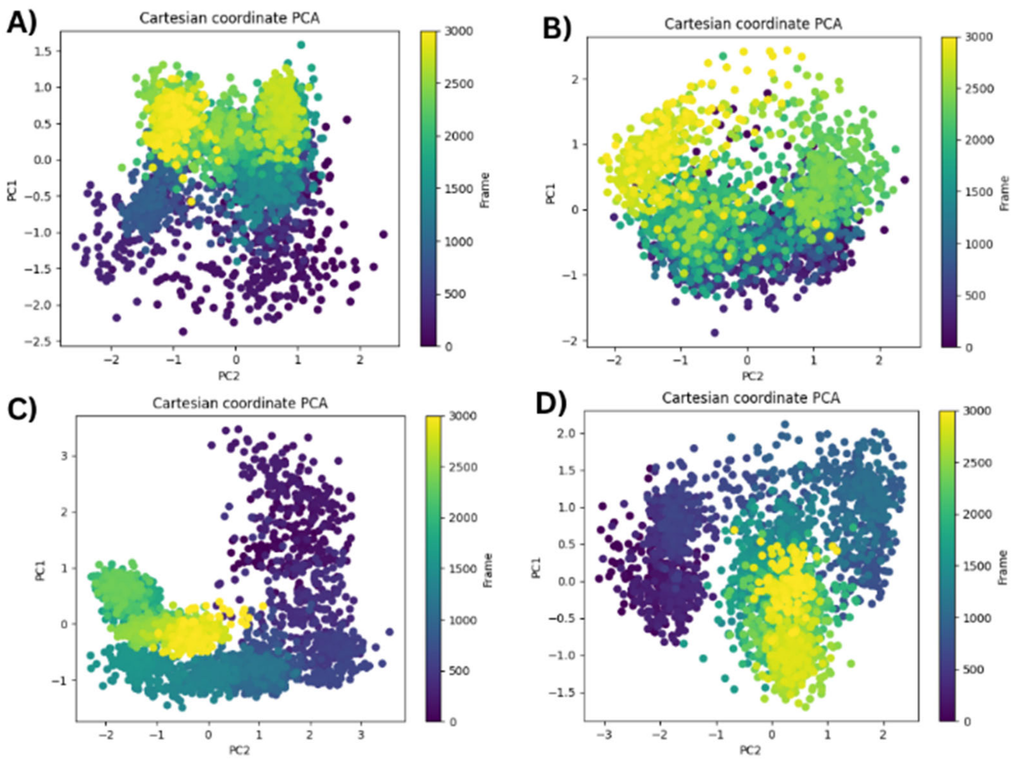

Figure S1. *Two-dimensional projection of the conformational ensemble of the four complexes onto PC1–PC2. A) 2RKU–myricetin, B) 2RKU–isorhamnetin, C) 2RKU–quercetin, D) 2RKU–kaempferol; colour encodes simulation frame.*

S2.3. Convergence of the essential subspace

Two complementary diagnostics were used to test whether the essential subspace of each single trajectory is internally converged (Table S2). The cosine content of a principal component ranges from 0 to 1; values close to 1 indicate a diffusive, random-walk-like mode characteristic of undersampling, whereas values close to 0 indicate a well-defined, sampled collective motion [11,12]. Three of the four complexes show low cosine content in their leading modes (≤0.18), and only quercetin retains a partially diffusive PC1 (0.62), consistent with its broader projection. In parallel, the root mean square inner product (RMSIP) between the subspace obtained from the growing interval [0, t] and that of the full trajectory reaches a stable plateau of 1.00 in all four systems, and the RMSIP between four consecutive time blocks remains above the conventional 0.7 criterion (0.71–0.79) [13,14]. Together, these results suggest that the orientation of the essential subspace is converged within the 300 ns, which is the property underlying the structural stability reported in the main text.

Table S2. *Convergence diagnostics of the essential subspace per complex: cosine content of the leading principal components, running RMSIP (start → plateau) and mean RMSIP between four consecutive time blocks.*

Complex	Cosine content PC1 / PC2 / PC3	Running RMSIP (start → plateau)	Block RMSIP (mean, 4 blocks)
**Isorhamnetin**	0.08 / 0.05 / 0.02	0.80 → 1.00	0.79
**Kaempferol**	0.18 / 0.05 / 0.02	0.72 → 1.00	0.74
**Myricetin**	0.02 / 0.03 / 0.01	0.78 → 1.00	0.73
**Quercetin**	0.62 / 0.18 / 0.07	0.77 → 1.00	0.71

S2.4. Reproducibility across independent complexes

To assess reproducibility beyond a single run, the four complexes were projected onto a common reference subspace and the pairwise RMSIP between their essential subspaces was computed (Figure S2). Because the four simulations differ in ligand, initialisation and thermalisation, they constitute independent realisations of the same receptor. All off-diagonal values lie between 0.72 and 0.80 (mean 0.77 ± 0.03), indicating that the dominant collective motions are recovered consistently across independent complexes rather than being an artefact of a particular trajectory [13,14]. This inter-complex consistency provides, in the absence of formal replicas, an internal check of reproducibility and is the dynamic counterpart of the conserved binding mode described in the main text.

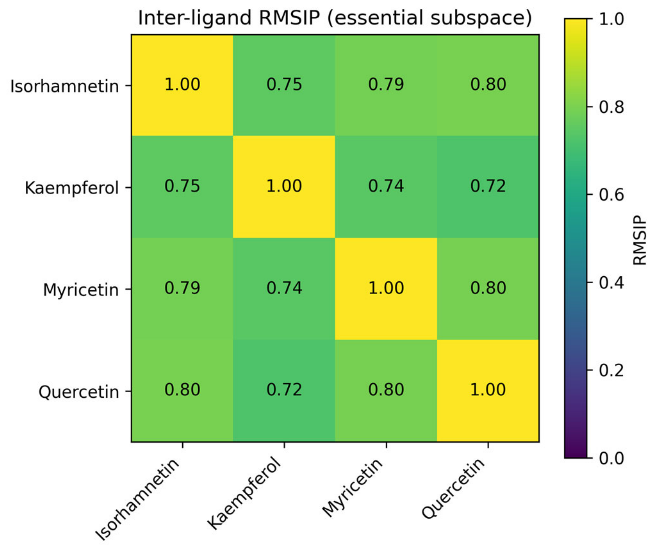

Figure S2. *Pairwise RMSIP among the essential subspaces of the four independent PLK-1–flavonoid complexes on a common reference (1 = identical, 0 = orthogonal).*

S2.5. Statistical reliability of the principal-component time series

The statistical quality of the principal-component time series was quantified rather than assessed visually (Table S3). After automated equilibration detection [15], the integrated autocorrelation time (g) and the corresponding production effective sample size, N_eff = (N − t0)/g,

were computed for PC1 and PC2, and the true statistical error of PC1 was estimated by block averaging [16]. The effective sample sizes are modest (N_eff(PC1) = 18–120) and the autocorrelation times long, with block-averaged PC1 errors of 2.0–6.9 Å. These figures are reported explicitly: they indicate that a single 300 ns trajectory samples the slowest modes a limited number of times, which is why the present analysis is used to describe the direction and convergence of the collective motion (Tables S2, Figure S2) rather than to derive quantitative populations. The small block-averaged errors of PC1 are nonetheless consistent with the low, stable Cα-RMSD reported in the main text.

Table S3. *Statistical treatment of the principal-component time series: automated equilibration time t0, integrated autocorrelation time g, production effective sample size N_eff for PC1 and PC2, and block-averaged standard error of PC1.*

Complex	Equilibration t0 (PC1)	g (PC1)	N_eff prod. PC1 / PC2	Block-SEM PC1 (Å)
**Isorhamnetin**	0 ns	163	18 / 40	2.10
**Kaempferol**	228 ns	386	120 / 42	4.63
**Myricetin**	279 ns	136	66 / 93	2.00
**Quercetin**	269 ns	825	40 / 58	6.95

S2.6. Robustness of the conformational states

Because different clustering algorithms encode different definitions of a conformational state, the agreement among several methods is a more reliable test than any single algorithm. Four algorithms — K-means, Ward, GROMOS [17] and DBSCAN — were applied in the five-component subspace, and the inter-method agreement was quantified with the adjusted Rand index (ARI) and the normalised mutual information (NMI) (Table S4). The three partition-based methods return the same number of clusters and a moderate, consistent agreement (ARI 0.18–0.34; NMI 0.33–0.45), whereas the density-based DBSCAN collapses to a single dominant cluster in three of the four systems. Read jointly, this pattern suggests a continuous, single-dominant-basin landscape rather than a set of well-separated discrete states [18,19], which is consistent with the preserved globular fold and with the single-well free-energy landscapes of the main text. Clustering is therefore presented as an exploratory characterisation of macrostates rather than as a quantitative population partition.

Table S4. *Multi-method clustering in the five-component subspace: number of clusters selected by the silhouette criterion for each algorithm and inter-method agreement (ARI, NMI) among the partition methods.*

Complex	k (silhouette)	Clusters KM / Ward / GROMOS / DBSCAN	ARI	NMI
**Isorhamnetin**	6	6 / 6 / 6 / 1	0.18	0.33
**Kaempferol**	5	5 / 5 / 5 / 4	0.34	0.45
**Myricetin**	6	6 / 6 / 6 / 1	0.34	0.45

**Quercetin**	5	5 / 5 / 5 / 1	0.32	0.41

S2.7. Inter-ligand comparison and structure–activity link

Finally, the four complexes were compared on the common essential subspace to place each system in context (Table S5, Figure S2). Global flexibility, quantified by the covariance trace (the sum of the eigenvalues; larger values denote greater mobility), follows the order quercetin (541.0 Å²) > kaempferol (489.7 Å²) > isorhamnetin (417.0 Å²) > myricetin (358.5 Å²). The comparative free-energy landscapes (Figure S3) show that every complex explores a connected, single-dominant-basin region on identical axes. Notably, myricetin — the ligand with the most hydroxylated B ring and the most favourable MM-GBSA binding energy (main text, Section 2.5.6) — forms the most rigid complex, whereas quercetin is the most flexible and shows the only diffusive leading mode. This monotonic association between B-ring hydroxylation, hydrogen-bond anchoring, complex rigidity and binding strength appears to provide an independent, dynamics-based line of support for the structure–activity interpretation of the main text, in line with comparable kinase–inhibitor analyses [20,21].

Taken together, the analyses in this section suggest that the single-trajectory essential dynamics of the four complexes are internally converged (Tables S2), reproducible across independent complexes (Figure S2) and describable as a single dominant conformational basin (Table S4, Figure S3), while the modest effective sample sizes (Table S3) delimit their interpretation to these directional and comparative properties. These observations are consistent with, and reinforce, the structural stability, binding-mode conservation and MM-GBSA affinity ordering presented in the main text.

Table S5. *Inter-ligand comparison on the common essential subspace: global flexibility (covariance trace, ascending), mean RMSIP to the other three ligands, and degree of B-ring hydroxylation.*

Complex	Covariance trace (Å²)	Mean RMSIP vs. others	B-ring –OH (count)
**Myricetin**	358.5	0.78	3
**Isorhamnetin**	417.0	0.78	1 (+ 3′-OMe)
**Kaempferol**	489.7	0.74	1
**Quercetin**	541.0	0.77	2

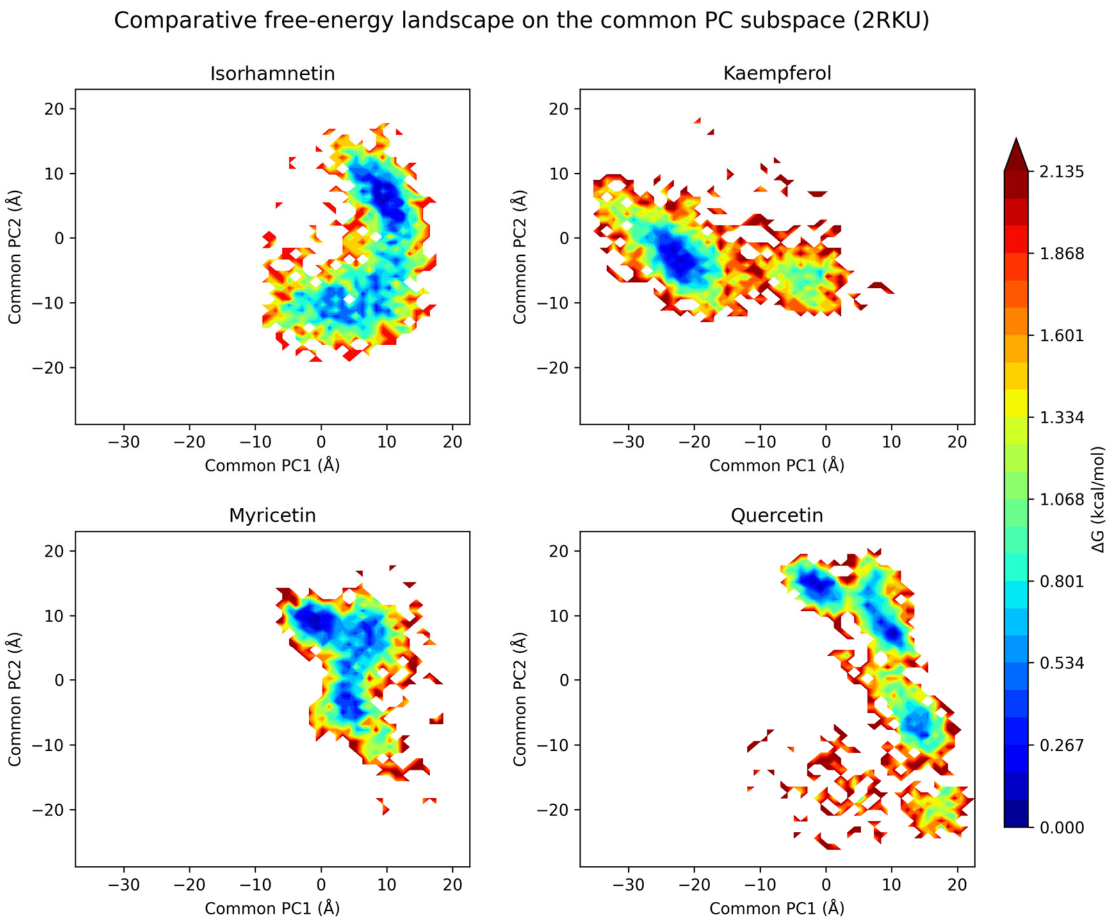

Figure S3. *Comparative free-energy landscapes of the four complexes on the common essential subspace (PC1–PC2). Each complex explores a connected, single-dominant-basin region.*

References

1. Houson, H.A.; Wright, B.D.; Fernandez, S.R.; Buss, T.; White, S.L.; Cederstrom, B.; Omweri, J.M.; McConathy, J.E.; Schnitzer, J.E.; Lapi, S.E. PET Imaging Expedites Detection of Aberration in the Humanization of an Annexin A1 Targeting Antibody. *Pharmaceuticals* **2025**, *18*, 295, doi:10.3390/ph18030295.
2. Kalin, S.; Comert Onder, F. Discovery of potential RSK1 inhibitors for cancer therapy using virtual screening, molecular docking, molecular dynamics simulation, and MM/GBSA calculations. *J. Biomol. Struct. Dyn.* **2025**, *43*, 1424-1444, doi:10.1080/07391102.2023.2291830.
3. Ansari, W.A.; Rab, S.O.; Saquib, M.; Sarfraz, A.; Hussain, M.K.; Akhtar, M.S.; Ahmad, I.; Khan, M.F. Pentafuhalol-B, a Phlorotannin from Brown Algae, Strongly Inhibits the PLK-1 Overexpression in Cancer Cells as Revealed by Computational Analysis. *Molecules* **2023**, *28*, 5853, doi:10.3390/molecules28155853.
4. Alhadrami, H.A.; Sayed, A.M.; Hassan, H.M.; Alhadrami, A.H.; Rateb, M.E. Molecular insights and inhibitory dynamics of flavonoids in targeting Pim-1 kinase for cancer therapy. *Front. Pharmacol.,* **2024**, *Volume 15 - 2024*, doi:10.3389/fphar.2024.1440958.

5. Lu, X.; Zhang, L.; Sun, S.; Wang, S.; Shi, Y. Structural Insights into Water-mediated Interactions in Kinase-Inhibitor Complexes: Implications for Drug Design. *Sci Rep* **2020**, *10*, 1408.
6. Zhang, J.; Zhang, L.; Wang, J.; Ouyang, L.; Wang, Y. Polo-like Kinase 1 Inhibitors in Human Cancer Therapy: Development and Therapeutic Potential. *J. Med. Chem.* **2022**, *65*, 10133-10160, doi:10.1021/acs.jmedchem.2c00614.
7. Chiappa, M.; Petrella, S.; Damia, G.; Broggini, M.; Guffanti, F.; Ricci, F. Present and Future Perspective on PLK1 Inhibition in Cancer Treatment. *Front Oncol* **2022**, *12*, 903016, doi:10.3389/fonc.2022.903016.
8. Lešnik, S.; Jukić, M.; Bren, U. Unveiling polyphenol-protein interactions: a comprehensive computational analysis. *J. Cheminform.* **2025**, *17*, 50, doi:10.1186/s13321-025-00997-3.
9. Zhang, Q.; Zhu, W.; Cai, Y.; Meng, Y.; Tan, W.; Li, J.; Lu, X.; Du, H. Structure-Based Virtual Screening Discovers Potent PLK1 Inhibitors with Anticancer Activity. *J. Chem. Inf. Model.* **2026**, *66*, 1050-1066, doi:10.1021/acs.jcim.5c02253.
10. Moradi, S.; Nowroozi, A.; Aryaei Nezhad, M.; Jalali, P.; Khosravi, R.; Shahlaei, M. A review on description dynamics and conformational changes of proteins using combination of principal component analysis and molecular dynamics simulation. *Comput. Biol. Med.* **2024**, *183*, 109245, doi:https://doi.org/10.1016/j.compbiomed.2024.109245.
11. Hess, B. Convergence of sampling in protein simulations. *Phys. Rev. E* **2002**, *65*, 031910, doi:10.1103/PhysRevE.65.031910.
12. Palma, J.; Pierdominici-Sottile, G. Fortuitous Correlations in Molecular Dynamics Simulations: Their Harmful Influence on the Probability Distributions of the Main Principal Components. *ACS Omega* **2024**, *9*, 20488-20501, doi:10.1021/acsomega.4c01515.
13. Amadei, A.; Ceruso, M.A.; Di Nola, A. On the convergence of the conformational coordinates basis set obtained by the essential dynamics analysis of proteins' molecular dynamics simulations. *Proteins: Struct., Funct., Bioinf.* **1999**, *36*, 419-424, doi:https://doi.org/10.1002/(SICI)1097-0134(19990901)36:4<419::AID-PROT5>3.0.CO;2-U.
14. Ormeño, F.; General, I.J. Convergence and equilibrium in molecular dynamics simulations. *Commun. Chem.* **2024**, *7*, 26, doi:10.1038/s42004-024-01114-5.
15. Chodera, J.D. A Simple Method for Automated Equilibration Detection in Molecular Simulations. *J. Chem. Theory Comput.* **2016**, *12*, 1799-1805, doi:10.1021/acs.jctc.5b00784.
16. Flyvbjerg, H.; Petersen, H.G. Error estimates on averages of correlated data. *J. Chem. Phys.* **1989**, *91*, 461-466, doi:10.1063/1.457480.
17. Daura, X.; Gademann, K.; Jaun, B.; Seebach, D.; van Gunsteren, W.F.; Mark, A.E. Pept. Folding: When Simul. Meets Exp. *Angewandte Chemie International Edition* **1999**, *38*, 236-240, doi:https://doi.org/10.1002/(SICI)1521-3773(19990115)38:1/2<236::AID-ANIE236>3.0.CO;2-M.
18. González-Alemán, R.; Hernández-Castillo, D.; Caballero, J.; Montero-Cabrera, L.A. Quality Threshold Clustering of Molecular Dynamics: A Word of Caution. *J. Chem. Inf. Mod.* **2020**, *60*, 467-472, doi:10.1021/acs.jcim.9b00558.
19. Schultze, S.; Grubmüller, H. Time-Lagged Independent Component Analysis of Random Walks and Protein Dynamics. *J. Chem. Theory Comput.* **2021**, *17*, 5766-5776, doi:10.1021/acs.jctc.1c00273.
20. Ahmad, B.; Batool, M.; Ain, Q.U.; Kim, M.S.; Choi, S. Exploring the Binding Mechanism of PF-07321332 SARS-CoV-2 Protease Inhibitor through Molecular Dynamics and Binding Free Energy Simulations. *Int. J. Mol. Sci.* **2021**, *22*, 9124, doi:10.3390/ijms22179124.
21. Alqahtani, A. Integrating AI-Based Clustering, Molecular Dynamics, and Binding Energy Analysis to Elucidate Conformational Dynamics and Binding Selectivity in Extracellular Signal-Regulated Kinases 1 and 2 Complexes. *Bioinform. Biol. Insights* **2026**, *20*, 11779322261427124, doi:10.1177/11779322261427124.
